# Supplementary material for: Exon definitive regions for MPC1 microexon splicing and its usage for splicing modulation
Source: Mol Ther Nucleic Acids. 2023 Jan 25;31:398–410. doi: 10.1016/j.omtn.2023.01.010 (PMC9929638; doi:10.1016/j.omtn.2023.01.010)
Supplement: Document S1. Figures S1–S4 and Tables S1 and S2 [file mmc1.pdf]

OMTN, Volume *31*

Supplemental information

Exon definitive regions for *MPC1* microexon splicing and its usage for splicing modulation

Eunjin Koh, Daye Shin, and Kyung-Sup Kim

Table_S1

Primers used in reporter construct generation

primer	Sequence (5' to 3')
Int31-199-s	GAAACTGTCATTAAACTGACTTGAGTTCTTATTAGAAATAATATTACTTGATC
Int31-199-r	GTAATATTATTTCTAATAAGAACTCAAGTCAGTTTAATGACAGTTTCCCCCAAA
Int31-96-s	GGAAACTGTCATTAAACTGACTTGGTTGCAGTAACCTAATAAGACCAAAATCAT
Int31-96-r	TGGTCTTATTAGGTTACTGCAACCAAGTCAGTTTAATGACAGTTTCCCCCAAA
Int31-67-s	GGAAACTGTCATTAAACTGACTTGTTCCATGTCTGTCCTGTCTGAACTTCGGTG
Int31-67-r	GTTCAGACAGGACAGACATGGAACAAGTCAGTTTAATGACAGTTTCCCCCAAA
Int31-46-s	CTGTCATTAAACTGACTTGTTTGATTTTTGTTTCTTTTTTTTCCATGTCTGTCCTGTCT
Int31-46-r	GACATGGAAAAAAAAGAAACAAAAATCAAACAAGTCAGTTTAATGACAGTTTCCCCCAAA
Int31-25-s	GAAACTGTCATTAAACTGACTTGCTTCGGTGACTCTTCACAAAATAGTACGGTA
Int31-25-r	CTATTTTGTGAAGAGTCACCGAAGCAAGTCAGTTTAATGACAGTTTCCCCCAAA
ex-4d4-FOR	GTGACTCTTCACAAAATAGGTAAGAAAATTATTTCCATCCATTT
ex-4d4-REV	ATGGAAATAATTTTCTTACCTATTTTGTGAAGAGTCACCGAAGT
ex-3d4-FOR	GTGACTCTTCACAAAATAGGGTAAGAAAATTATTTCCATCCATTT
ex-3d4-REV	AAATGGATGGAAATAATTTTCTTACCCTATTTTGTGAAGAGTCAC
ex-2d4-FOR	GTGACTCTTCACAAAATAGTGGTAAGAAAATTATTTCCATCCATTT
ex-2d4-REV	ATGGAAATAATTTTCTTACCACTATTTTGTGAAGAGTCACCGAAGT
ex-1d4-FOR	GTGACTCTTCACAAAATAGTCGGTAAGAAAATTATTTCCATCCATTT
ex-1d4-REV	ATGGAAATAATTTTCTTACCGACTATTTTGTGAAGAGTCACCGAAGT
b-globin-intron-fwd	TTATTTGTCAGATTGTAAGTGTTGCAGTAACCTAATAAGACC
b-globin-intron-rev	AAAAGTGATTAGAGAAAGTTCTTACCGTACTATTTTGTGAAGA
EF1a-intron-fwd	GCACCTCGATTAGTTCTCGAGTTGCAGTAACCTAATAAGACC
EF1a-intron-rev	GACGTACTCCAAAAGCTCGATCTTACCGTACTATTTTGTGAAGA
GPD-20-fwd	CTTTAGGCTGCAAGGACCCGGCCCAGGTGAGAGGGGCACAGAGGCAGCTATGG
GPD-20-rev	CCATAGCTGCCTCTGTGCCCCTCTCACCTGGGCCGGGTCCTTGCAGCCTAAAG
GPD-8-fwd	CCCTCCTCACTTTAGGCTGCAAGGTGAGAGGGGCACAGAGGCAGCTATG
GPD-8-rev	CATAGCTGCCTCTGTGCCCCTCTCACCTTGCAGCCTAAAGTGAGGAGGG
GPD-5-fwd	AGCCTTGCCCCCTCCTCACTTTAGGCAAGGTGAGAGGGGCACAGAGGCAGCTATG
GPD-5-rev	CATAGCTGCCTCTGTGCCCCTCTCACCTTGCCTAAAGTGAGGAGGGGGCAAGGCT
GPD-3ss-fwd	TAAGCCCAGGAGTTTGAGTCCAGTTGCAGTAACCTAATAAGACC
GPD-113-3ss-rev	TCCCTGGGCCGGGTCCTTGCAGCCTATTTTGTGAAGAGTCACCGAAG
GPD-20-3ss-rev	CTCACCTGGGCCGGGTCCTTGCAGCCTATTTTGTGAAGAGTCACCGAAGTT
GPD-8-3ss-rev	CTCTGTGCCCCTCTCACCTTGCAGCCTATTTTGTGAAGAGTCACCGAAGT

GPD-5-3ss-rev	TGCCTCTGTGCCCCTCTCACCTTGCCTATTTTGTGAAGAGTCACCGAAG
GPD-113-35ss-fwd	GGAGCCTTAAAGGTAAGAAAATTATTTCCATCCATTTCAGGTGAGGAGAAGGCCCCAAAG
GPD-113-35ss-rev	TTCTCCTCACCTGAAATGGATGGAAATAATTTTCTTACCTTTAAGGCTCCACAGATCTCT
GPD-20-35ss-fwd	CCCGGCCCAGGTAAGAAAATTATTTCCATCCATTTCAGGTGAGGAGAAGGCCCCAAAG
GPD-20-35ss-rev	TTCTCCTCACCTGAAATGGATGGAAATAATTTTCTTACCTGGGCCGGGTCCTTGCAGC
GPD-8-35ss-fwd	AAATAGGCTGCAAGGTAAGAAAATTATTTCCATCCATTTCAGGTGAGGAGAAGGCCCCAAAG
GPD-8-35ss-rev	TTCTCCTCACCTGAAATGGATGGAAATAATTTTCTTACCTTGCAGCCTATTTTGTGAAGAGT
GPD-5-35ss-fwd	ACAAAATAGGCAAGGTAAGAAAATTATTTCCATCCATTTCAGGTGAGGAGAAGGCCCCAAAG
GPD-5-35ss-rev	TTCTCCTCACCTGAAATGGATGGAAATAATTTTCTTACCTTGCCTATTTTGTGAAGAGTCAC
GPD-8-3ss-5ssmut-fwd	CTTCACAAAATAGGCTGCAAGGTAAGAGGGGCACAGAGGCAGCTATG
GPD-8-3ss-5ssmut-rev	CATAGCTGCCTCTGTGCCCCTCTTACCTTGCAGCCTATTTTGTGAAG

Table_S2

Primers used in quantitative real-time RT-PCR and conventional RT-PCR

primer	Sequence (5' to 3')
E1E2-s	ATTTCCGGGACTACCTCATGAGTACG
AS	GCAATGGGAAGACCCCAGTT
E1E3-s1	GATTTCCGGGACTACCTCATGAGCACT
GPD1-I-s	TTCTGTGAGACAACCATTGGCAAG
GPD1-I-as	GAACTTGTGGCCGTTTACGT
GPD1-S-s	TTCTGTGAGACAACCATTGAATGT
GPD1-S-as	GAACTTGTGGCCGTTTACGT
UE-s	GGACTATGTCCGAAGCAAGGAT
EGFP-as	TGAACAGCTCCTCGCCCTT
EGFP1-s	GTGAGCAAGGGCGAGGAGCTGTTC
EGFP1-as	TTGCCGGTGGTGCAGATGAACTTCA
E3-as	GCAATGGGAAGACCCCAGTT
I3-s	TCCTGTCTGAACTTCGGTGA
SMN_678_S	GCTATCATACTGGCTATTATATGGGTTT
SMN_68_S	GCTATCATACTGGCTATTATATGGAAATG
SMN2ex8 as	CGCTTCACATTCCAGATCTG

Figure S1

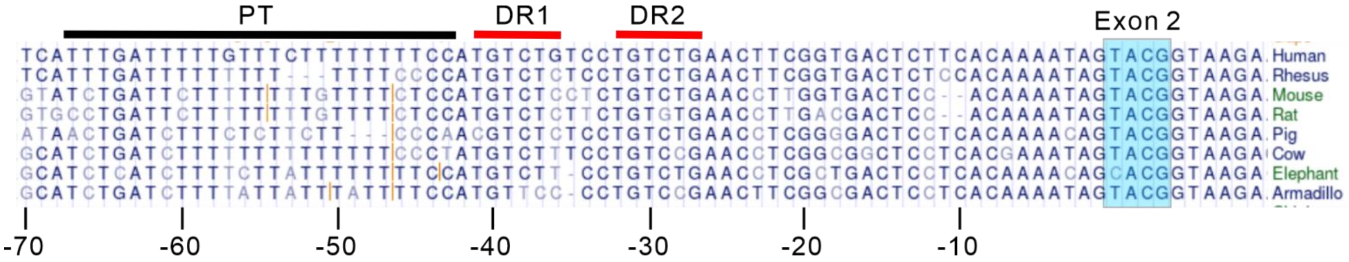

Figure S1. Sequence homology between mammalian species around the *MPC1* microexon

Figure S2

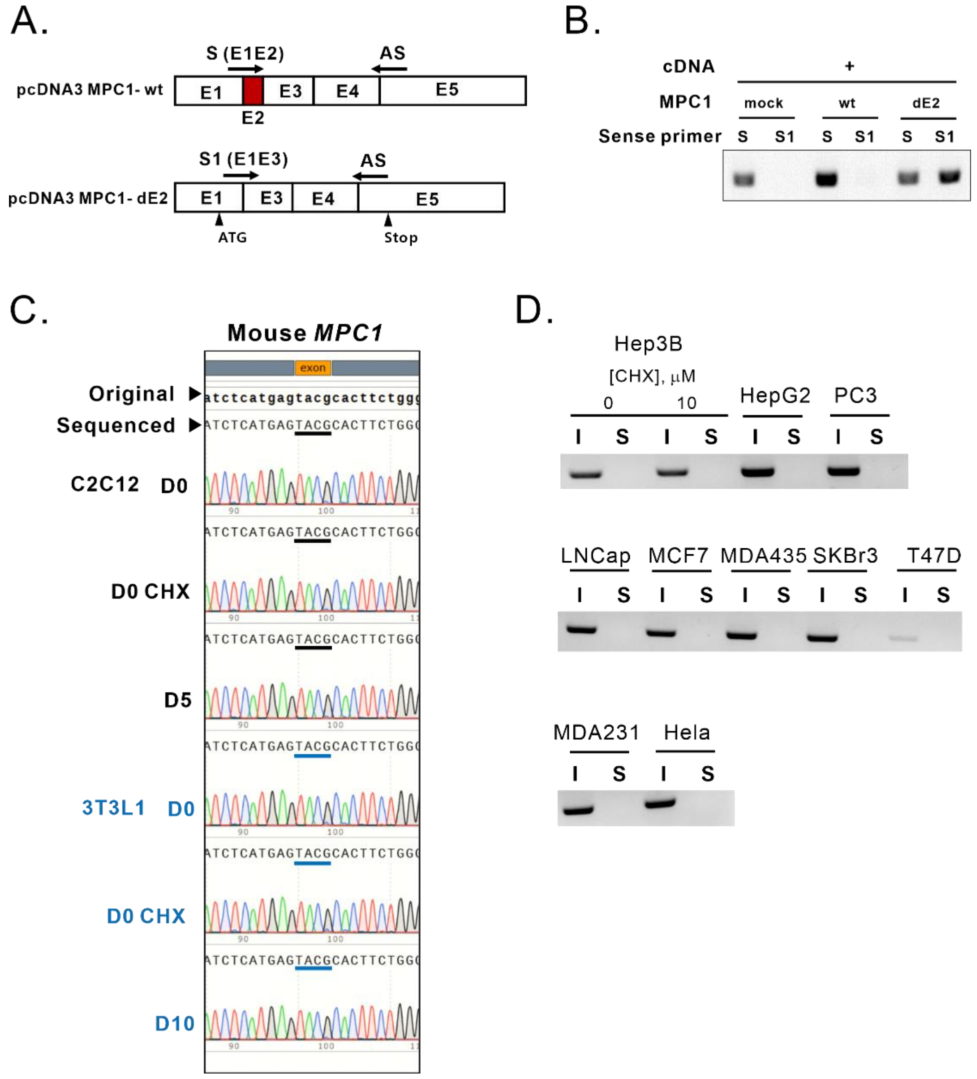

Figure S2. Detection of *MPC1* microexon-included and -skipped isoforms. (A) The primers were designed to detect the microexon skipped isoforms. The specificity was verified with the *MPC1* cDNA construct of wild-type (wt) and that of exon2 deleted (dE2) forms. (B) Agarose gel electrophoresis of RT-PCR products. Mixture of the one μl of one-fifth diluted cDNA synthesized from total RNA of 786-O cells and 10 pg of each mock, pcDNA3-MPC1-wt and pcDNA3-MPC1-dE2 were utilized as the template for PCR reactions. (C) Sequencing analysis of RT-PCR products of the mouse MPC1 mRNA prepared from C2C12 and 3T3L1 cell lines (D0, undifferentiated; D5 or D10 indicate 5 days or 10 days differentiated, respectively). Ten μM of cycloheximide (CHX) was treated for 6 hrs. Primers used in RT-PCR are mMPC-s 5'- ccacagcggtgtcatctgtc -3' and mMPC1-as 5'- gtaccgctatgcagatggcc-3'. (D) Detection of human MPC1 microexon-included or -skipped mRNA by RT-PCR in various cell lines. 'I' and 'S' indicate microexon- included and -skipped, respectively.

Figure S3

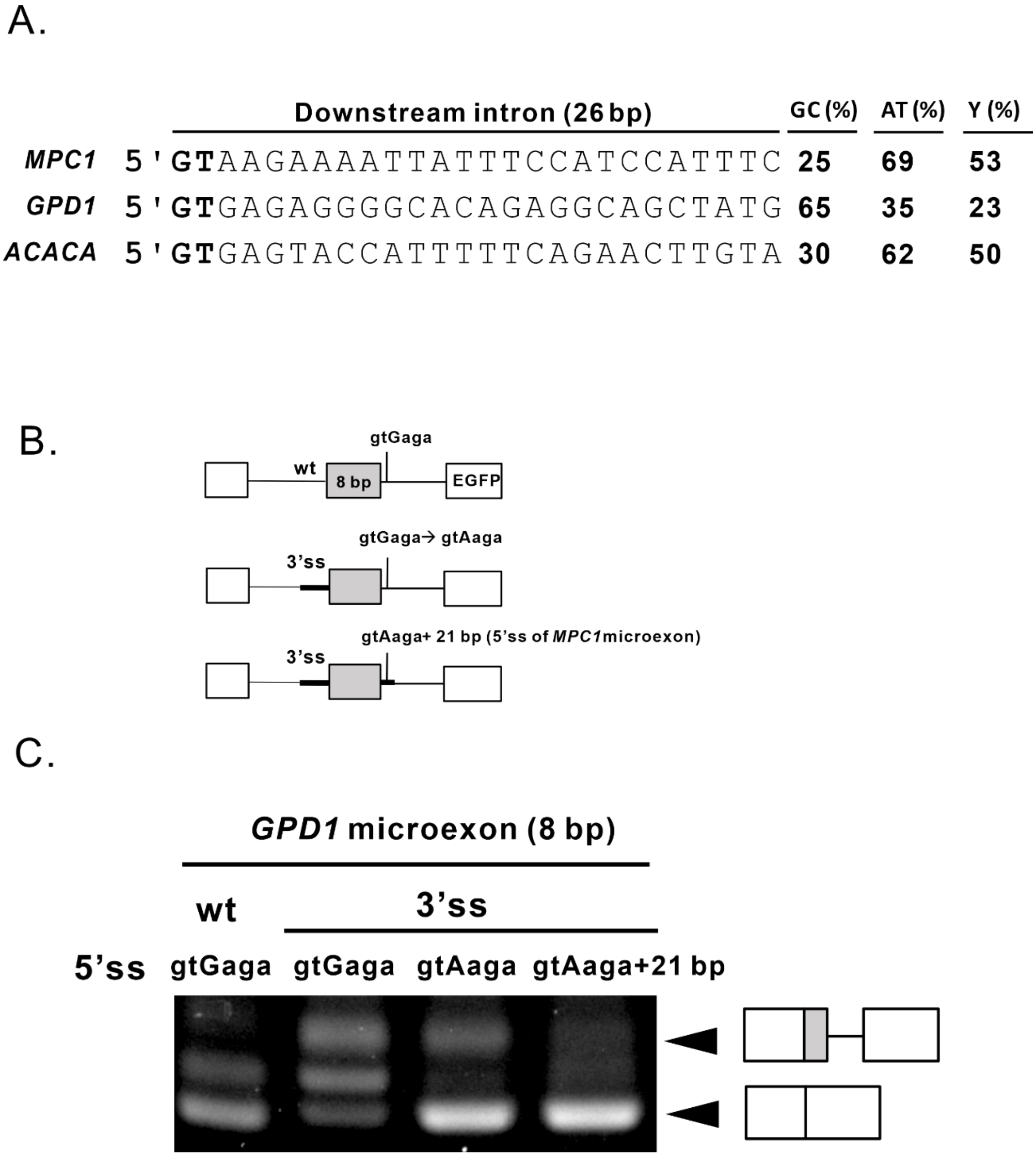

Figure S3. Cryptic GT usage is suppressed by 3'ss (96 bp) and 5'ss (25 bp) of *MPC1* microexon flanking introns. (A) Sequence alignment of 26 bp downstream intron of each microexon. Intron 2, intron 6, and intron 26 of *MPC1, GPD1*, and *ACACA*, respectively. Y; pyrimidines. (B) 5'ss of *GPD1* was mutated to that of *MPC1* (guGaga>guAaga) and was switched to 5'ss of *MPC1* microexon downstream intron (25 bp). (C) Agarose gel electrophoresis of RT-PCR products.

Figure S4

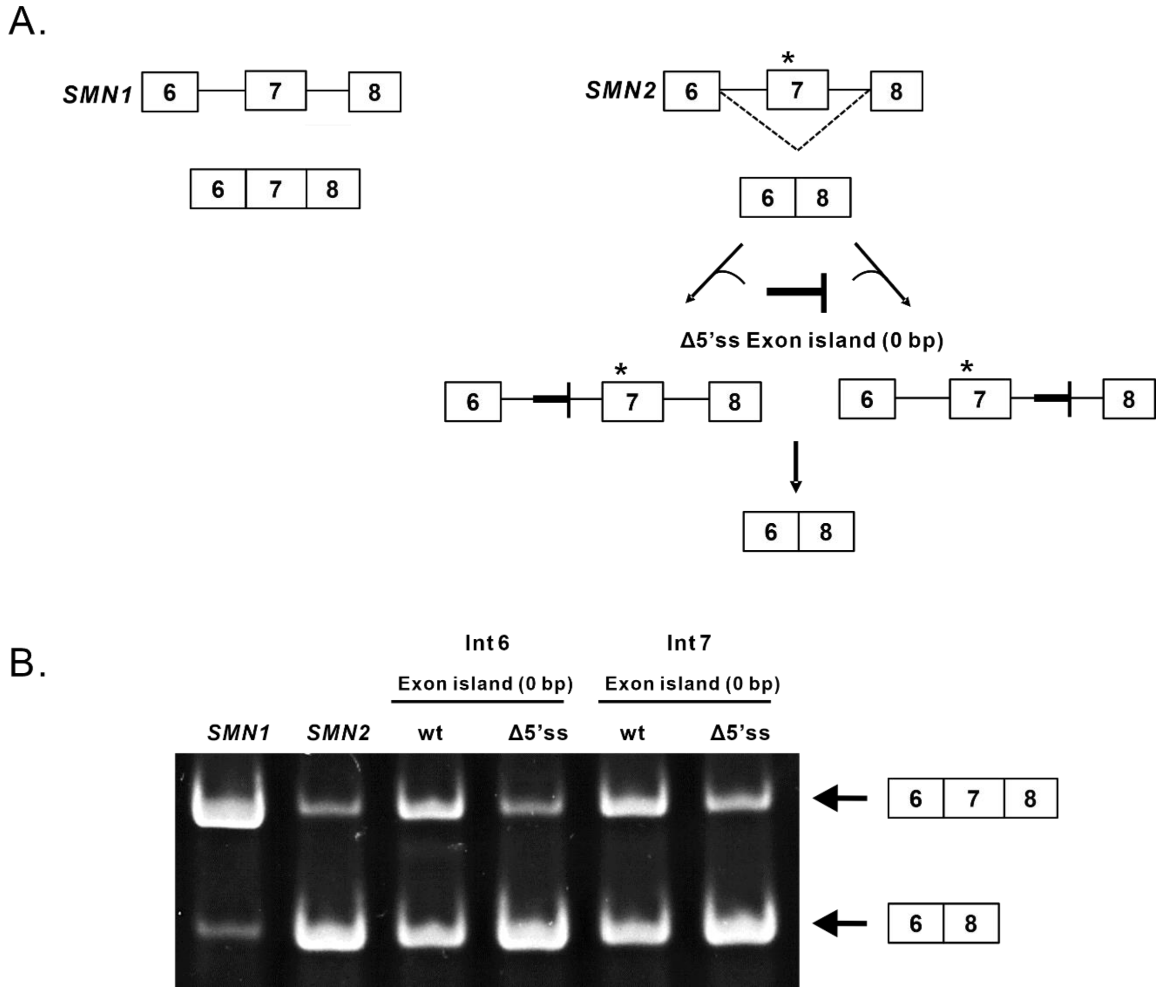

Figure S4. Insertion of the Δ5’ss exon island (0 bp) did not enhance exon 7 inclusion. (A) *SMN1* and *SMN2* minigene constructs were positioned between the SV40 promoter and *EGFP* gene in the pSV40-EGFP backbone. Asterisk (*) indicates C>T mutation. *SMN2* minigene vector was modified by the insertion of Δ5‘ss exon island (0 bp) into either intron 6 or intron 7. (B) Acrylamide gel electrophoresis of RT-PCR products amplified with sense and tisense primers bind to exon 6 and exon 8, respectively.
