# Supplementary material for: Exon definitive regions for MPC1 microexon splicing and its usage for splicing modulation
Source: Mol Ther Nucleic Acids. 2023 Jan 25;31:398–410. doi: 10.1016/j.omtn.2023.01.010 (PMC9929638; doi:10.1016/j.omtn.2023.01.010)
Supplement: Document S2. Article plus supplemental information [file mmc2.pdf]

Molecular Therapy
Nucleic Acids
Original Article

Exon definitive regions for *MPC1* microexon splicing and its usage for splicing modulation

Eunjin Koh,[1] Daye Shin,[1] and Kyung-Sup Kim[1]

[1]Department of Biochemistry and Molecular Biology, Institute of Genetic Science, Yonsei University College of Medicine, Seoul 03722, Korea

Alternative splicing of microexons (3–30 base pairs [bp]) is involved in important biological processes in brain development and human cancers. However, understanding a splicing process of non-3x bp microexons is scarce. We showed that 4 bp microexon of *mitochondrial pyruvate carrier1* (*MPC1*) is constitutively included in mRNA. Based on our studies with minigene and exon island constructs, we found the strong exon definition region in the proximal introns bordering *MPC1* microexon. Ultimately, we defined a nucleotide fragment from the 3′ss 67 bp of *MPC1* microexon to the 5′ss consensus sequence, as a core exon island, which can concatenate its microexon and neighboring exons by splicing. Furthermore, we showed that insertion of the core exon island into a target exon or intron induced skip the target exon or enhance the splicing of an adjacent exon, respectively. Collectively, we suggest that the exon island derived from *MPC1* microexon modifies genuine splicing patterns depending on its position, thereby providing insights on strategies for splicing-mediated gene correction.

INTRODUCTION

Splicing of precursor mRNA (pre-mRNA) into mRNA is essential for gene expression in eukaryotic cells. Splicing is governed by many *cis-* and *trans*-elements, such as RNA-binding proteins (RBPs) and heterogeneous nuclear ribonuclear proteins (hnRNPs). Thus, its complexity is sufficient to explain that the misregulation of splicing leads to many abnormal cellular functions.[1] In fact, aberrant splicing has been implicated in many human diseases.[2]

In contrast to common exons with sizes of approximately 130 base pairs (bp),[3] microexons under 50 bp are easily skipped due to their short length and lack of exonic splicing enhancers (ESEs), as well as spatial limitations for spliceosome assembly at 3′ and 5′ splice sites (ss) to fulfill exon definition.[4–6] Furthermore, genomic mapping and annotation analysis showed that the number of constitutively spliced microexons markedly drops with exon size.[7–9] Moreover, there are alternatively spliced (AS) microexons that are used in particular tissues such as the brain, heart, muscle, and pituitary gland, although their relevance needs further investigation.[7,9] Recently, the function of microexons and their splicing processes have gained substantial attention because microexons exhibit important biological functions despite their small size. The misregulation of microexons is involved in abnormal brain development, autism, and various cancers.[9–15] Thus far, the regulatory mechanisms on microexon splicing have been studied with multiple of 3 bp (3x bp) microexons. RBPs, which regulate alternative splicing, are involved in microexon splicing in tissue and disease-specific manners. RBFOX and PTBP1 proteins act as enhancers and suppressors of microexon splicing, respectively.[9] SRRM4, a brain-specific RBP, stimulates the inclusion of microexon in the brain and various cancers.[10,12,15,16] QUAKING (QKI) plays important roles in microglia homeostasis by regulating alternative splicing of Rho GTPase pathway-related microexons.[14] Ubiquitously present RBPs, Srsf11, and Rnps1 identified by genome-wide CRISPR-Cas9, preferentially regulate neuronal microexons.[13] In addition, *cis* elements that reside in flanking introns are important regulators of microexon splicing. Replacement of purines in the upstream polypyrimidine tract with pyrimidines can recover the skipped microexons,[5] and intronic splicing enhancer, "GGGGCUG," located downstream of 5′ss activates microexon inclusion through SF1 binding.[17] The sequence at the branchpoint is also involved in microexon partial inclusion.[18] Despite the importance of microexons in abnormal brain development and cancers, the mechanism by which microexons are spliced and the functions of amino acid sequences encoded by these microexons are largely unknown.

In contrast to 3x bp exons, aberrant splicing of non-3x exons can lead to the formation of premature termination codon (PTC) resulting in degradation of transcripts through nonsense-mediated decay (NMD) followed by compromised protein levels in the cell.[19] In the same light, a recent study demonstrated that the inclusion of a 20 bp microexon of *Bak1* is a target of NMD that contributes to the reduction of Bak1 during brain development.[11] However, studies on the splicing mechanism and functions of non-3x microexons are still greatly in need.

MPC1 is a component of the mitochondrial pyruvate carrier (MPC) complex, which mediates important metabolic processes by transporting cytosolic pyruvate into the mitochondria.[20,21] Since the expression of MPC1 is essential for stabilizing the MPC complex, the regulation of *MPC1* mRNA levels has been investigated, including

Received 17 October 2022; accepted 20 January 2023;
https://doi.org/10.1016/j.omtn.2023.01.010.

Correspondence: Kyung-Sup Kim, Department of Biochemistry and Molecular Biology, Institute of Genetic Science, College of Medicine, Yonsei University, Seoul 03722, Korea.
E-mail: kyungsup59@yuhs.ac

 © 2023 The Author(s).
This is an open access article under the CC BY license (http://creativecommons.org/licenses/by/4.0/).

in our recent study.[22] In particular, exon 2 of *MPC1* is a very short non-3x bp microexon (4 bp) flanked with long introns on both sides. However, no study on *MPC1* microexon has been conducted to our knowledge.

Here, we demonstrated that a 4 bp microexon of *MPC1* is constitutively included during splicing through exon definition at its proximal flanking introns. We have defined a nucleotide fragment from the upstream 67 bp of *MPC1* microexon to the downstream 6 bp, as a core exon island with a strong exon definition enough to splice various microexon sequences into the final mRNA. We also presented promising gene-editing actions of this defined exon island based on various genetic models.

RESULTS

Microexon of *MPC1* is constitutively included during the splicing process

We investigated the human genome and counted microexons under 27 bp in length (Figure 1A). The number of microexons of each length was counted based on the values of Chr_Accession, Exon_Start, Exon_End, and Exon_length_bp from the Gene_Table.xlsx spreadsheet file provided by Piovesan et al.[23] Most microexons under 12 bp have 3x bp microexons (77%). Noticeably, there are very limited numbers of non-3x bp microexons under 12 bp. Two genes (*GRK6* and *SEPT7*) have the shortest microexons with 2 bp in the human genome. Based on the information of transcript variants in the NCBI gene web site (https://www.ncbi.nlm.nih.gov/gene), microexon in *GRK6* is found in minor transcript at the second to last exon, which produces the C-terminal variant. *SEPT7* has AS microexon located in 5′UTR, which does not affect amino acid sequence. Four and 5 bp microexons are found in eight genes (*TNNI1*, *MPC1*, *SPINK2*, *DCTD*, *KSR1*, *TMEM237*, *SPARCL1*, and *SEPT7*). Microexons of *DCTD* and *SPARCL1* were AS in the 5′UTR in minor transcripts, not affecting the amino acid sequence. *SEPIN7* has AS microexons at the second exon generating the alternative start codon resulting in several N-terminal variants. Microexons of the remaining genes (*TNNI1*, *MPC1*, *KSR1*, and *TMEM237*) and 7 bp microexon of *MUSK* are in the coding region and did not show skipped variants in the NCBI gene bank, so they were tested in this experiment. In addition, it is observed that the microexon and its proximal intron sequences of *MPC1* are highly conserved for cross-species (Figure S1), and those of the four genes we have tested (*TNNI1*, *KSR1*, *TMEM237*, and *MUSK*) also showed high homology across species.

Alternative splicing of microexons contributes to isoform production; however, aberrant splicing of non-3x microexons can cause a frameshift, resulting in PTC formation followed by defects in gene expression through NMD.[19] We amplified the region where microexons are in the transcripts of the five genes and directly sequenced RT-PCR products. As shown in Figure 1B the sequences of each microexon from five genes exhibited no overlapped peak by alternative spliced transcripts, indicating that no microexons of these genes were skipped in the cell lines we tested.

Next, we reconfirmed whether the 4 bp *MPC1* microexon is constitutively spliced. To exclude the possibility that microexon-skipped transcripts may be targets of NMD, 786-O cells were pre-treated with NMD inhibitor, cycloheximide (CHX), and transcripts were examined by RT-PCR (Figure 1C). Noticeably, there is no detectable microexon-skipped transcript even in the presence of CHX. These results suggest that the reason why microexon-skipped transcripts are not detected is due to the absolute splice-in of the microexon, not because of its clearance by NMD. In addition, the same results were observed when *MPC1* was transcriptionally enhanced by the overexpression of PGC-1α.[22] The same experiment was done in several other cell lines derived from mice and humans, and it was confirmed that the microexon was spliced almost completely (Figures S2C and S2D). Taken together, we could conclude that the microexon of *MPC1* is highly conserved in mRNA under normal and transcriptionally active states.

3′ss of intron 1 of *MPC1* is critical for the inclusion of its microexon

To assess the potential role of flanking introns as *cis* elements for *MPC1* microexon splicing, we generated a variety of *MPC1* minigene constructs (Figure 2A). All the constructs were transfected into HeLa cells, and total RNA prepared to perform quantitative real-time PCR. *MPC1* microexon minigene construct harboring the *MPC1* genome spanning exon 1 to exon 3, shortened by removing the middle part of introns (MPC1-wt). The upstream or downstream intron of exon 2 microexon was swapped with a foreign intron (*MPC1* intron 3), which has well-conserved splice sites, to make Swap-1/3 or Swap-2/3, respectively. As shown in Figure 2B, the Swap-1/3 completely lost microexons during splicing, while Swap-2/3 produced a considerable amount of mRNA harboring microexons, indicating that upstream introns are critical for the splicing-in of the *MPC1* microexon. Therefore, we next constructed intronic hybrid mutants, which the 5′ or 3′ region of intron 1 is replaced with the 5′ and 3′ region of intron 3, respectively, named Hybrid-3/1 and Hybrid-1/3. Notably, Hybrid-3/1 stimulated microexon inclusion, whereas Hybrid-1/3 failed microexon splicing, indicating that the 3′ss region of the intron 1 is critical for microexon splicing.

Next, to determine the minimal 3′ss region of intron 1 required for *MPC1* microexon splicing, 329 bp of intron 1 in Hybrid-3/1 were serially deleted, as depicted in Figure 2D (top). The inclusion of microexon was maintained until the 3′ss region of the upstream intron was removed up to 67 bp. However, the microexon was markedly lost when the 3′ region was reduced to 46 bp. These results indicate that 67 bp of the 3′ region of the upstream intron is a minimal region required for the splicing of *MPC1* microexon. As denoted in Figure 2D (bottom), 67 bp of the 3′ region of the upstream intron has a pyrimidine-rich region between −67 and −43 (22 pyrimidines out of 25). The further deletion to −23 bp completely abolished the splicing, where direct repeat (“TGTCTG”) is located. It would be further study to investigate about RBP binding or RNA secondary structure at the 67 bp minimal region that shows very similar sequences between species (Figure S1).

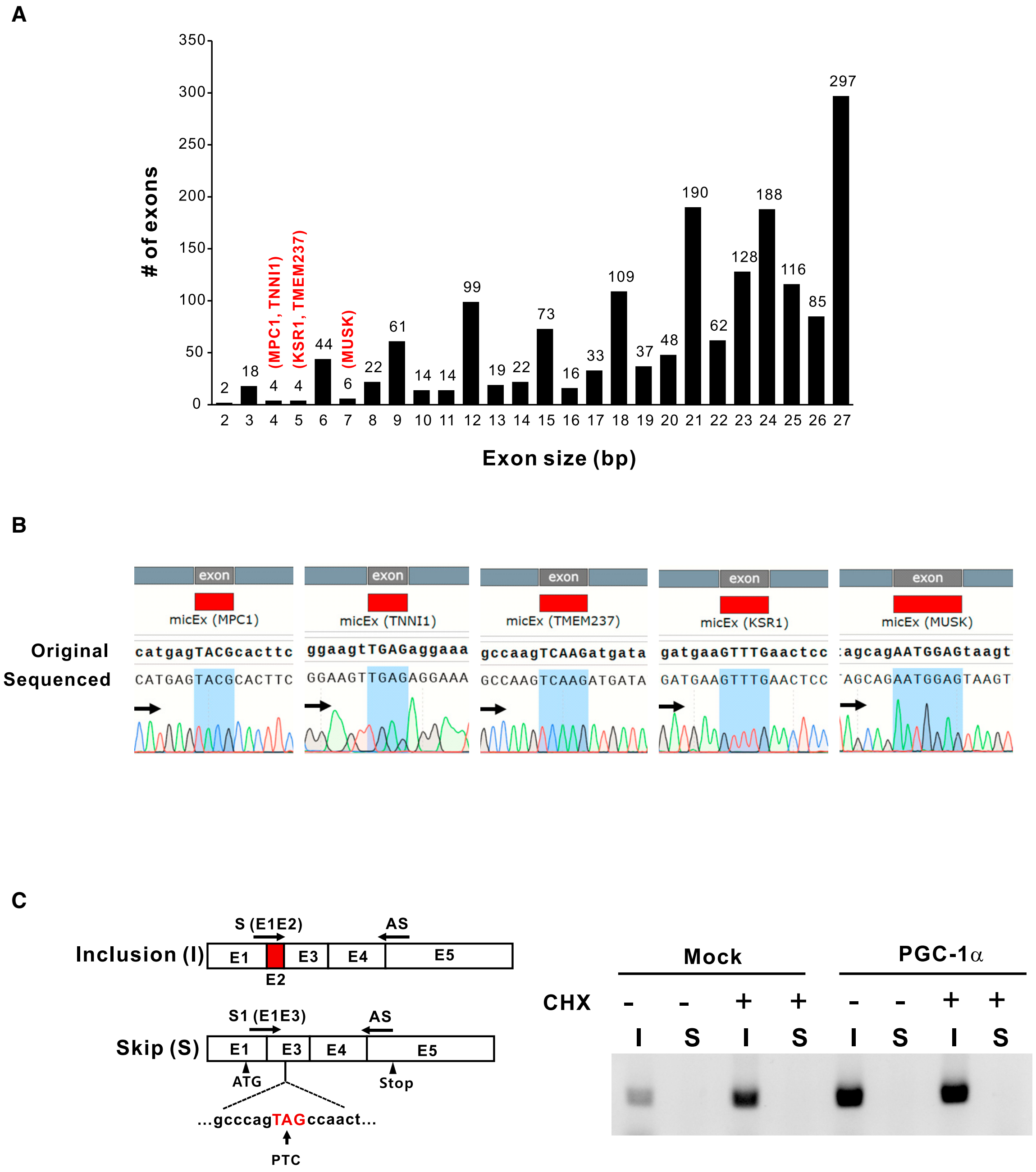

Figure 1. Microexon of *MPC1* is constitutively included in mRNA during the splicing process

(A) Analysis of microexons less than 27 bp in length. The numbers of microexons were counted based on the Gene_table.xlsx provided by Piovesan et al.[23] (B) Comparison of mRNA reference sequences from NCBI vs. those of RT-PCR amplified products. The regions of microexons from *MPC1* (4 bp), *TNNI1* (4 bp), *TMEM237* (5 bp), *KSR1* (5 bp), and *MUSK* (7 bp) are shaded (light blue). (C) RT-PCR analysis of *MPC1* mRNA. "I" and "S" indicate microexon-included and skipped mRNA, respectively. Arrows indicate the locations of primers used for the amplification of "I" and "S" (see Table S2). The specificity of the primers was validated (Figures S1A and S1B); 50 μM of cycloheximide (CHX) was treated for 6 h before total RNA extraction. Parental 786-O cells and 786-O cells with lentiviral stable expressed PGC-1α were used.

If the exon island is defined as a DNA fragment containing the exon and its surrounding intron sequences, which has a strong exon definition enough to concatenate neighboring exons by splicing, we investigated that *MPC1* microexon with its flanking intron sequences could play a role as exon island. To evaluate the exon island activity, the reporter vector was constructed, in which an upstream exon containing ATG start codon, intron 3 of *MPC1*, and a downstream exon were sequentially placed between the SV40 promoter and the EGFP

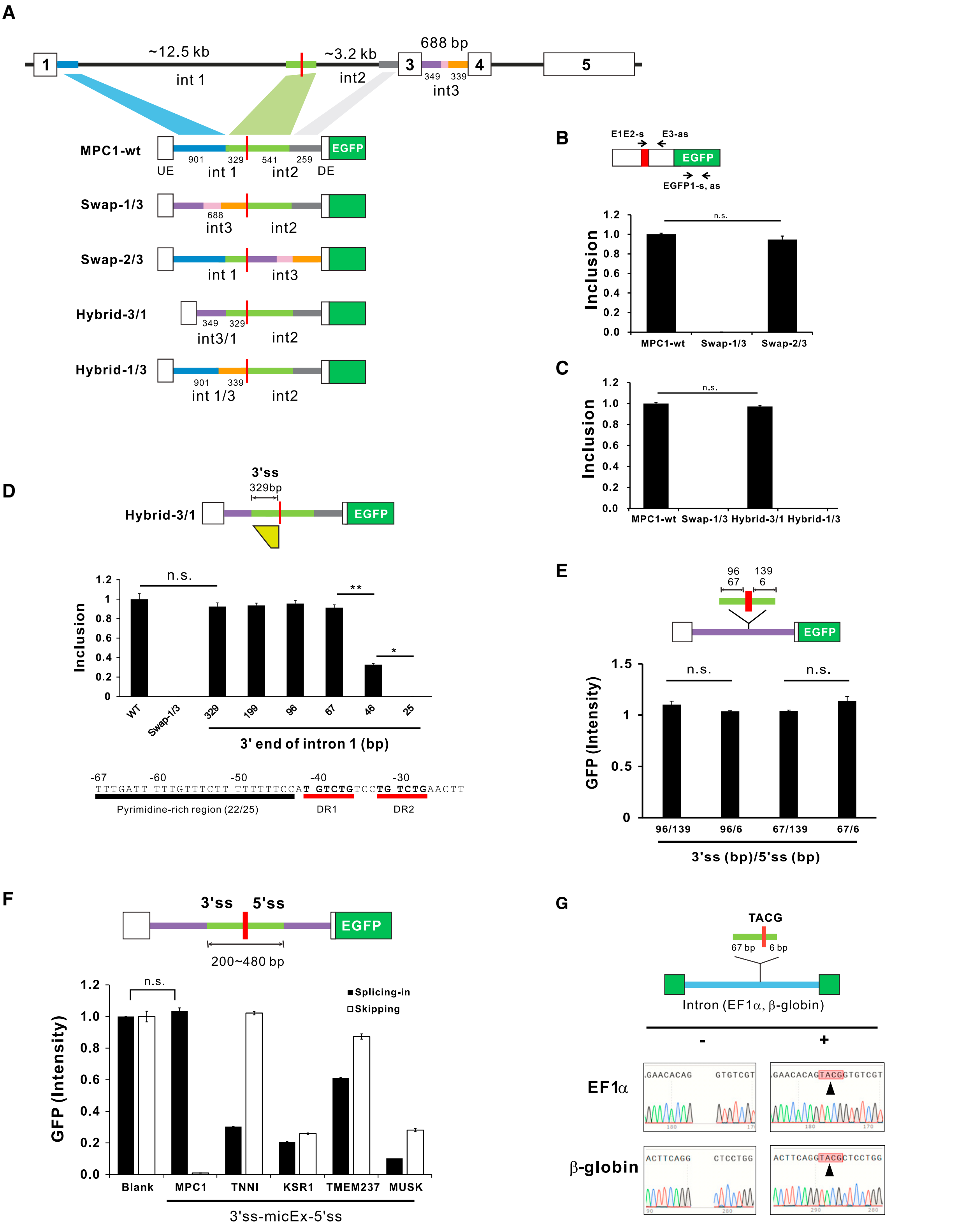

(legend on next page)

gene. *MPC1* microexon with its flanking intron sequences (light green) were placed in the middle of intron of the reporter vector as shown in Figure 2E. The 96 bp and 67 bp upstream introns along with 139 bp downstream intron showed full splicing activity as Hybrid-3/1 (67 bp) construct. Noticeably, no loss of splicing activity was observed until the downstream intron was reduced to 6 bp, the consensus 5′ss sequence in the intron. Based on these results, we could conclude that the 67 bp 3′ss of the upstream intron and 6 bp 5′ss of the downstream intron were sufficient for exon definition recognizing 4 bp ("TACG") as an exon.

The fact that non-3x microexons of four genes (*TNNI1, KSR1, TMEM237,* and *MUSK*) were constitutively conserved in endogenous mRNA intrigued us to explore whether their proximal flanking introns also accurately splice respective microexons. Reporter constructs of each microexon were designed in two forms, one expresses EGFP when the microexon is spliced in, and the other expresses EGFP when the microexon is skipped (Figure 2F). Therefore, the EGFP intensity of *MPC1* "splicing-in" reporter was the same as that of "blank," whereas its skipping reporter did not show any activity, indicating that the 3′ss and 5′ss of *MPC1* microexon (239 bp) led to the complete inclusion of microexons. On the contrary, nucleotide fragments of *TNNI1* (468 bp), *KSR1* (409 bp), *TMEM237* (477 bp), and *MUSK* (418 bp) are noticeably ineffective in microexon splicing-in and showed significant levels of microexon skipping, meaning that they might require other elements in addition to the proximal introns around the microexon for maximal inclusion of their microexons during splicing.

Next, we tried to ascertain whether the 3′ss (67 bp) and 5′ss (6 bp) of *MPC1* microexon could act as the effective exon island in different genomic contexts. Therefore, the 77 bp nucleotide fragment of *MPC1* core exon island was inserted into the middle of the first intron of *EF1α* and the second intron of rabbit *HHB2*; both are commonly used in conventional expression vectors, such as pEF1α-EGFP and pSG5-EGFP, respectively (Figure 2G). RT-PCR products were sequenced to confirm whether the 4 bp of microexon was spliced into the final mRNA. Remarkably, 4 bp microexon sequences were precisely sequenced without overlapping with skipped transcripts. Thus, we could conclude that the core exon island from 67 bp 3′ss to 6 bp 5′ss of *MPC1* microexon is sufficient for the exon definition of *MPC1* microexon in our experimental settings.

Splicing process is maintained until *MPC1* microexon length is reduced to zero

In the human genome, we could not find the microexon with a length of 1 bp, although this might come from the recent technical limitation. These facts raised the question of whether the splicing efficiency of the *MPC1* microexon could be preserved when the length of the microexon is shortened to less than 4 bp. Therefore, we generated *MPC1* microexon minigene constructs as illustrated in Figure 3A. Since the constructs with 4-, 2-, and 1-bp microexons were designed to express EGFP in frame when its corresponding microexon is correctly spliced into the final mRNA their splicing activities were evaluated by EGFP fluorescence intensity, as described in materials and methods. Compared with 4 bp (wt) MPC1 microexon, shortened 2 bp or 1 bp microexon showed no marked difference in their splicing activity, which indicates that these microexons were successfully spliced in mRNA (Figure 3B). RT-PCR and sequencing analysis confirmed that 4-, 2-, and 1-bp microexon sequences were included in the mRNA (data not shown).

In addition, it was intriguing to examine whether the splicing occurs when all the exon sequence is removed (0 bp) or when the consensus of 3′ss and 5′ss were compromised (−2 bp) (Figure 3A). As shown in Figure 3C, there was no noticeable difference of the spliced transcripts level in three minigene constructs, indicating that their splicing successfully proceeded in all constructs. To further investigate whether the splicing of the RNA from these constructs is processed with two steps at the junction of the upstream and downstream intron or both introns are removed at once, the lariats generated during the splicing process were examined with total RNA prepared from HeLa cells transfected with minigene constructs (Figure 3D). We first analyzed the level of lariat "b," which originated from the downstream intron (Figure 3A). Four bp minigene construct produced lariat "b" as expected, but −2 bp minigene construct in which 5′ss and 3′ss is

Figure 2. Inclusion of the microexon of *MPC1* is facilitated by the 3′ splice site of intron 1

(A) Schematic diagram explains the structure of *MPC1* minigene constructs used in experiments. MPC1-wt minigene contains *MPC1* microexon and its adjacent exon and shortened flanking introns. Swap −1/3 and 2/3 constructs have intron 3 (688 bp) instead of intron 1 or intron 2, respectively. Hybrid-3/1 and 1/3 constructs are generated by replacing the 5′ or 3′ regions of intron1 with those of intron 3 (purple or orange, respectively). All constructs have the following downstream sequences of EGFP and poly-A after exon 3. (B) and (C) *MPC1* microexon splicing activities. The level of the microexon-included mRNA was evaluated by quantitative real-time RT-PCR using E1E2-s and E3-as. Real-time RT-PCR values of the EGFP region by EGFP1-s and EGFP1-as were used as the total transcript level for normalization of the spliced-in of microexon (see Table S2 for primer sequences). (D) Intron 1 3′ end was serially deleted from Hybrid-3/1 construct (top). The level of microexon inclusion was measured by quantitative real-time PCR. The values were normalized in the same way as in (B) and (C). Sequence from −67 bp to −28 bp upstream of microexon was depicted (bottom). The pyrimidine tract and the direct repeat of "TGTCTG" are denoted as black and red lines. (E) and (F) The level of microexon inclusion was measured by EGFP fluorescence with whole-cell extracts of HeLa cells. For internal control of transfection efficiency, mCherry expression vector was co-transfected. EGFP intensities were divided by mCherry expression level. (E) The constructs were generated by inserting the fragments harboring 4 bp MPC1 microexon and its adjacent 3′ss and 5′ss in different lengths into intron 3 (purple) of the reporter vector (top). (F) Exon island activities of various microexons. The microexon islands of *MPC1* (239), *TNNI1* (468 bp), *KSR1* (409 bp), *TMEM237* (477 bp), and *MUSK* (418 bp) were amplified by PCR and inserted into the middle of intron in the reporter construct. (G) The 3′ss (67 bp) and 5′ss (6 bp) directed 4 bp *MPC1* microexon into mRNA. The 77 bp-length nucleotide fragments harboring a 4 bp *MPC1* microexon was inserted in the intron of either pEF1 α-EGFP or pSG5-EGFP plasmids. Constructs were transfected into the HeLa cells, followed by RT-PCR and sequence analysis. The inserted sequences of 4 bp, TACG, are shaded in red. Data are representative of three experiments. *$p < 0.05$ and **$p < 0.01$ by Student's two-tailed t test. All bar graphs are plotted as mean ± SE.

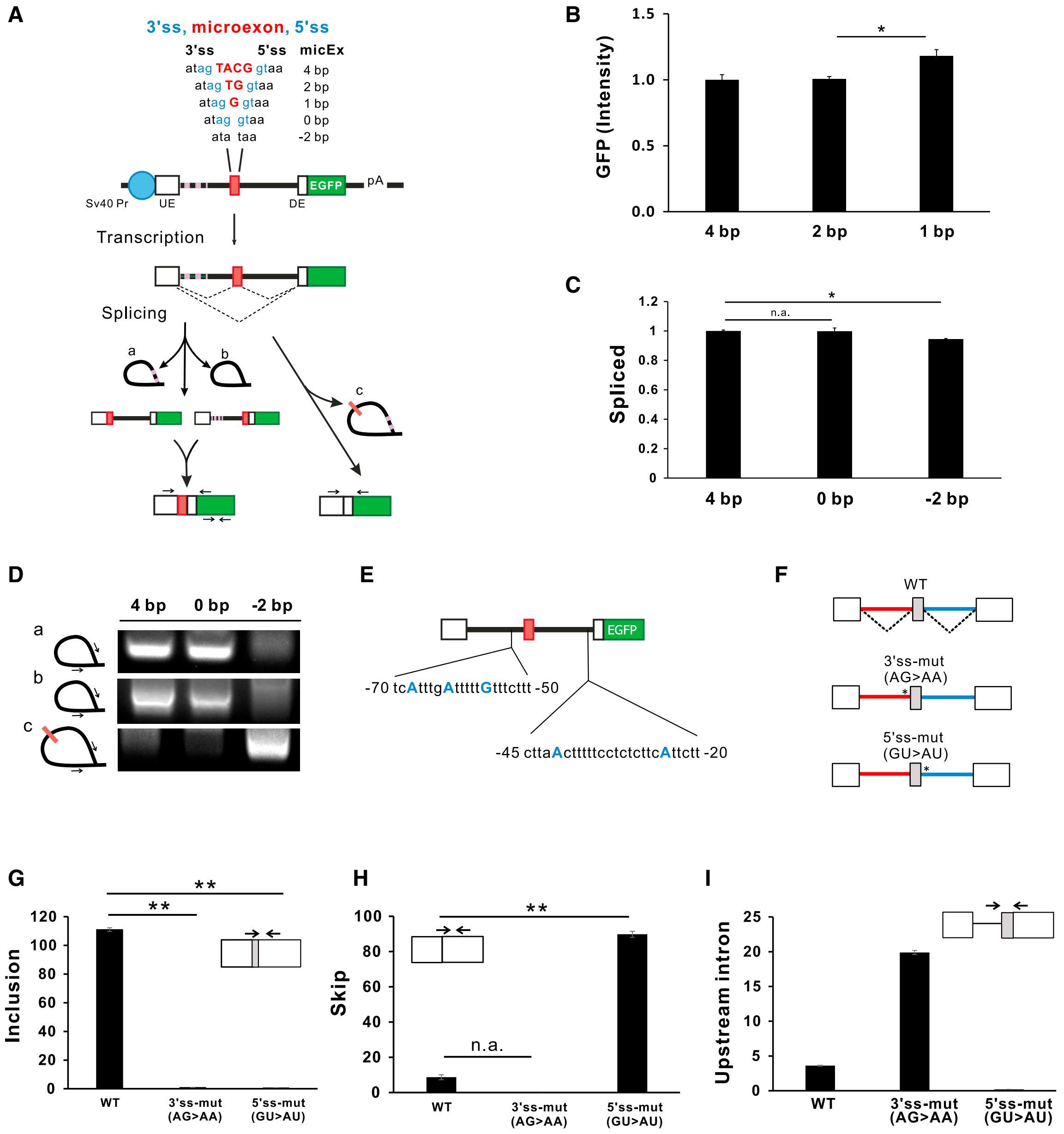

Figure 3. *MPC1* microexon splicing efficiently occurs when its length is shortened to 2, 1, and 0 bp

(A) *MPC1* minigene constructs containing various lengths of microexons. The structure of MPC1 minigene is same as MPC1-wt in Figure 2A except the microexon sequences (capital letters). The dotted line within intron 1 indicates a high GC region. The absence of exon and disruption of splicing sites are depicted as 0 and −2 bp. All constructs were designed to express EGFP when the microexon is correctly spliced into the final mRNA. (B) Splicing activities of *MPC1* minigene constructs containing microexons of various lengths. EGFP intensities were measured with whole-cell extracts of HeLa cells transiently transfected with each minigene construct. Values are normalized by the level of mCherry fluorescence expression considering transfection efficiency. (C) Comparison of the quantified spliced products among 4, 0, −2 bp minigene constructs by real-time RT-PCR using primers UE-s and EGFP-as (Table S2). The real-time RT-PCR values of the EGFP region were used for normalization of the spliced-in of microexon. (D) Agarose gel analysis of lariats by RT-PCR. Lariats a and c were amplified from Hybrid-3/1 minigene-transfected cells and lariat b from MPC1-wt. The lariats derived from *MPC1* minigene construct are depicted as “a,” “b,” and “c” in (A). Each lariat derived from 4 bp, 0 bp, −2 bp minigene construct was amplified by PCR and analyzed by agarose gel as described in the materials and methods. (E) The sequence around branch points in 3′ss of intron 1 and 2. Branchpoints (blue capital letters) are determined by sequencing 20 plasmids cloned from amplified lariat. The major branch sites of intron 1 were A at −68 and −64 and minor site was G at −57. The major branchpoints of intron 2 were A at −41 and −25. (F) *MPC1* minigene constructs of splice sites of 3′ss and 5′ss mutated to AG>AA and GU>AU, respectively. (G) to (I) Real-time RT-PCR analysis of transcripts synthesized with total RNA of HeLa cells transiently transfected with reporter constructs. Primers shown as arrows are E1E2-s and E3 primers for (G), E1E3-s1 and E3-as primers for (H), and I3-s and E3-as are used in (I). Primer sequences are listed in Table S2. Data are representative of three experiments. All bar graphs are plotted as mean ± SE. *$p < 0.05$ by Student’s two-tailed t test.

abrogated did not. Next, we analyzed lariat “a” and lariat “c” by using the Hybrid3/1 minigene constructs (Figure 2A) because we had difficulties in PCR to amplify these two lariats where the high GC contents (70%–80%) at the upstream 5′ region (Figure 3A, dotted line). As expected, lariat “a” was produced from the 4 bp minigene construct but not from the −2 bp minigene construct. Only lariat “c” was detected in −2 bp minigene construct transfected cells indicating that its splicing occurred at once between 5′ss of upstream and 3′ss of the downstream intron. It is also noteworthy to observe that 0 bp minigene construct produced lariat “a” as well as lariat “b,” indicating that even in the absence of exon, each downstream and downstream intron were spliced through transesterification reactions mediated by their 5′ss and 3′ss. Collectively, these results suggest that splicing occurs regardless of the length of microexons, even in the absence of an exon, if 3′ss and 5′ss around *MPC1* microexon are intact.

Next, we explored the effect of the disruption of 3′ss or 5′ss on exon splicing. We prepared *MPC1* microexon minigene constructs with 3′ss and 5′ss disrupting mutations as 3′ss (AG>AA) or 5′ss (GU>AU) (Figure 3F). Both mutations completely inhibited the splicing of their microexons (Figure 3G). However, there was an apparent difference in their splicing patterns. In the case of 5′ss mutation, the skipping of microexons was markedly induced, like that observed when both 3′ss and 5′ss were compromised (Figure 3H). In contrast, 3′ss mutation led to the splicing of downstream introns resulting in intron 1 retention (Figure 3I). These results indicate that the *cis* elements of proximal upstream introns may act as splicing enhancers for downstream intron splicing, even when 3′ss is disrupted.

Splicing of reconstituted microexon is facilitated by 3′ss and 5′ss of *MPC1* microexon

The exon definitive action of 67 bp 3′ss and 6 bp 5′ss of *MPC1* microexon was further supported by the experiments with reconstituted microexon minigene constructs. Previous papers, shortening of constitutively included normal exon to less than 50 bp led to their complete exclusion during splicing.[5] Therefore, we prepared the minigene constructs containing normal size exon 25 (125 bp) of acetyl-CoA carboxylase (*ACACA*), or its reduced 8-bp-length microexon, and their splicing activities were evaluated (Figures 4A and 4B). As expected, the inclusion of reconstituted microexon was almost completely prevented in contrast with 125-bp-length normal exon. Given that the 3′ss region in the upstream intron of *MPC1* microexon is critical for 4 bp microexon splicing, it is speculated that this region stimulates the inclusion of reconstituted microexon. To test this prediction, the 3′ss region of an upstream intron of *ACACA* minigene construct was switched to 3′ss (96 bp) of *MPC1* microexon, and its splicing activity was investigated. Remarkably, the inclusion of 8 bp microexon was completely recovered by replacing its 3′ss with that of the *MPC1* microexon (Figure 4B). The electrophoresis and sequence analysis of the RT-PCR product confirmed the inclusion of *ACACA* reconstituted microexon in final mRNA (Figure 4C).

We further recapitulated these data with *glycerol-3-phosphate dehydrogenase 1* (*GPD1*) (Figure 4D). As shown in Figure 4E, all reconstituted microexons (20, 8, and 5 bp) from normal exon 5 (113 bp) were skipped during splicing. The agarose gel electrophoresis of RT-PCR revealed the expected sizes of exon-included or -skipped RT-PCR products, which were also confirmed by sequencing analysis (Figure 4F).

As in the case of *ACACA*, it was expected that switching of the 3′ss region of an upstream intron of all the *GPD1* minigene constructs with that of *MPC1* microexon would completely rescue their splicing activity (Figure 4G). As shown in Figure 4H, all the *GDP1*-microexon-3′ss constructs showed a marked increase in EGFP expression, indicating that 3′ss of *MPC1* greatly recovered the inclusion of reconstituted microexons. However, the agarose gel electrophoresis of RT-PCR products exhibited unexpected upper bands (marked as *) produced from each *GDP1*-microexon-3′ss, while *GPD1*-113-3′ss exhibited only one single band (Figure 4I). Interestingly, sequencing data revealed that lower bands contained each microexon spliced-in, whereas upper bands were aberrant transcripts spliced at downstream cryptic 5′ss depicted as “GT” in Figure 4I, indicating that cryptic 5′ss rather than genuine 5′ss was preferred when reconstituted microexons were spliced. These data led us to compare 5′ss regions (26 bp) of *MPC1*, *ACACA,* and *GPD1* microexons, and it turned out that the *GPD1* 5′ss region shows higher GC, lower AT, and lower pyrimidines than those of *ACACA* and *MPC1* (Figure S2A). Based on these observations, it is conceivable that downstream intron 5′ss of *MPC1* microexon may play a role in microexon splicing. As shown in Figure 4J, we generated various constructs with each exon flanked with 3′ss (96 bp) and 5′ss (27 bp) from *MPC1* microexon flanking introns (3’5′ss), followed by the measurement of splicing activities. Surprisingly, the splicing activity of microexons was greatly enhanced in 3′5′ss constructs (Figure 4K). Moreover, RT-PCR showed a single major product without cryptic splicing in all minigene constructs (Figure 4L). The sequencing analysis revealed that 113 bp and all microexons were included in the final mRNA. Collectively, these data indicate that both 3′ss and 5′ss of *MPC1* microexon exert stimulatory effects on *GPD1*-reconstituted microexon inclusion.

Exon island of *MPC1* microexon regulates the splicing process through its exon definitive action

The finding that the minimal region of *MPC1* microexon flanking introns acts as an exon definition prompted us to test whether it could modulate canonical splicing processes. As shown in Figure 5A, the length of microexon within the exon island was adjusted to 5 bp so that the reading frame could be maintained when the microexon replaces the target exon. Then, we observed the effect of the exon island (5 bp) on the splicing process when it is positioned in the middle of exon 5 (113 bp) of *GPD1*- and in the 5′ss intron 25 of *ACACA* minigene construct (Figures 5B and 5C, left). These constructs were introduced into HeLa cells, and total RNA was extracted to perform RT-PCR. Noticeably, a reduced size of the PCR band was observed when exon island (5 bp) was introduced at each target site (Figures 5B and 5C, right). Sequencing analysis revealed that the lower band of each group contained the 5 bp (“TAACG”), which

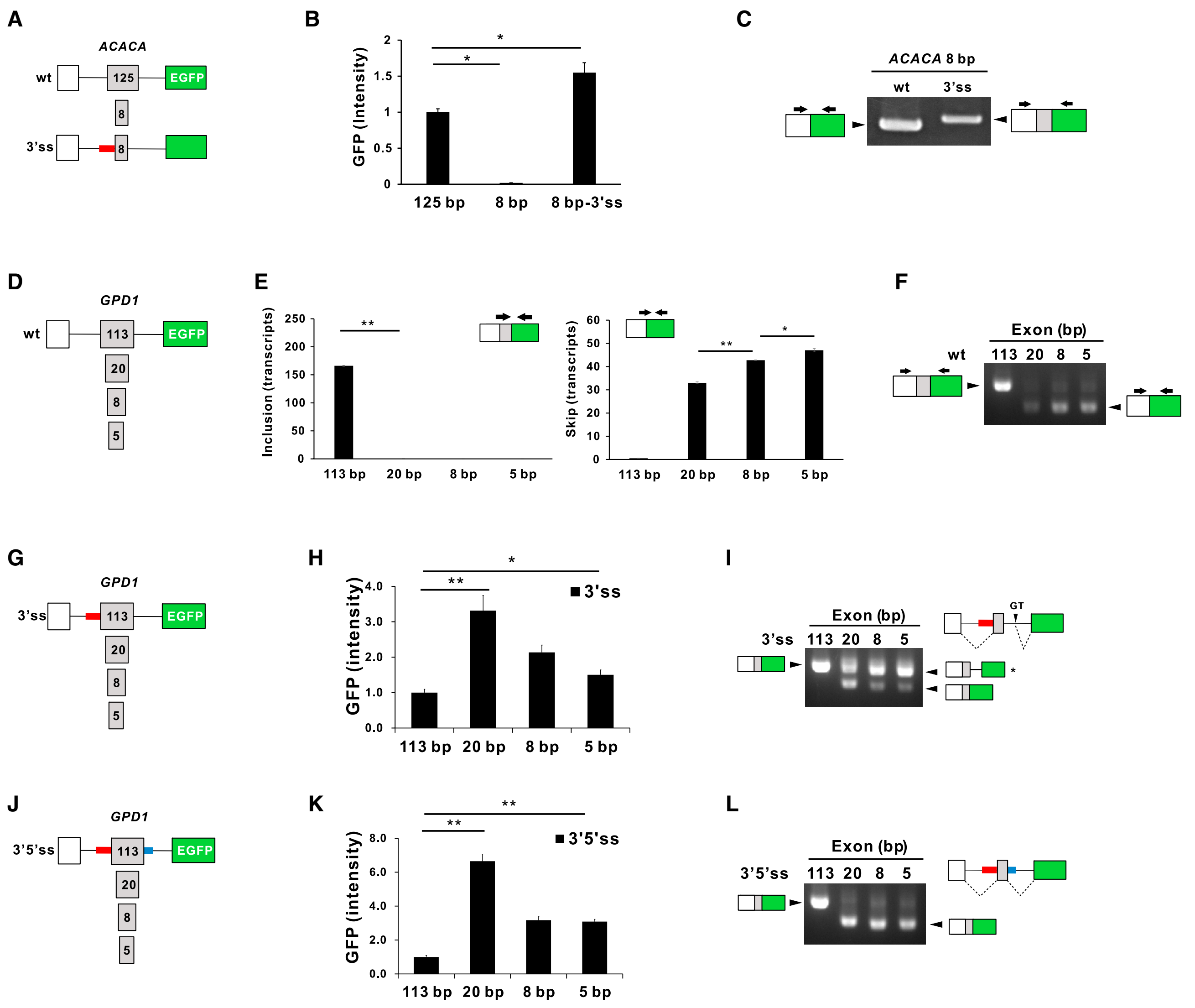

Figure 4. Splicing of reconstituted microexons is recovered by 3′ss and 5′ss of *MPC1* microexon

(A) Wild-type (wt) and 3′ss *ACACA* minigene constructs. The 3′ss of wt *ACACA* constructs was switched with the 3′ss 96 bp of *MPC1* intron 1. (B), (H), and (K) Splicing activities by measurement of GFP fluorescence. GFP intensities are normalized by mCherry expression level. (C) Acrylamide gel (8%) analysis of RT-PCR products to check whether the microexon is included or not. (D) *GPD1* minigene construct was generated by insertion of DNA fragment from the exon 4 to exon 6 including exon 5 (113 bp) of GPD1 gene between SV40 promoter and EFGP gene. Then, the length of exon 5 is shortened as indicated in gray boxes. (E) Quantification of exon inclusion and skipping of minigene constructs by real-time RT-PCR. (F), (I), and (L) Agarose gel analysis of RT-PCR products from mRNA transcribed from each minigene construct. HeLa cells 48 h after transfection. (G) The 3′ss *GPD1* minigene constructs. The 3′ss of *GPD1* intron 4 was switched with that of 3′ss 96 bp of *MPC1* intron 1. (J) The 3′5′ss *GPD1* minigene constructs. Both the 3′ss and 5′ss of *GPD1* intron 4 and intron 5 were replaced by those of *MPC1* intron 1 (3′ss 96 bp) and intron 2 (5′ss 27 bp), respectively. Data are representative of three experiments. *$p < 0.05$ and **$p < 0.01$ by Student's two-tailed t test.

derived from the exon island, indicating splicing occurred through the 3′ss and 5′ss within the exon island resulting in skipping of its target exon. These data demonstrate that the exon island of *MPC1* microexon could be used to force exon skipping while maintaining the reading frame.

Next, we tested the possibility that the exon island might stimulate neighboring exon splicing. We used the spinal muscular atrophy genetic model with SMN protein deficiency due to *SMN1* mutation. Since the *SMN2* is almost identical to *SMN1,* it would be expected to compensate for the SMN protein production. However, the nucleotide difference at exon 7 of *SMN2* skips exon 7 during splicing, which makes *SMN2* unable to produce the proper amount of SMN protein.[24]

Therefore, it was tempting to examine if the exon island of *MPC1* microexon could induce exon 7 inclusion in mRNA of *SMN2*. We

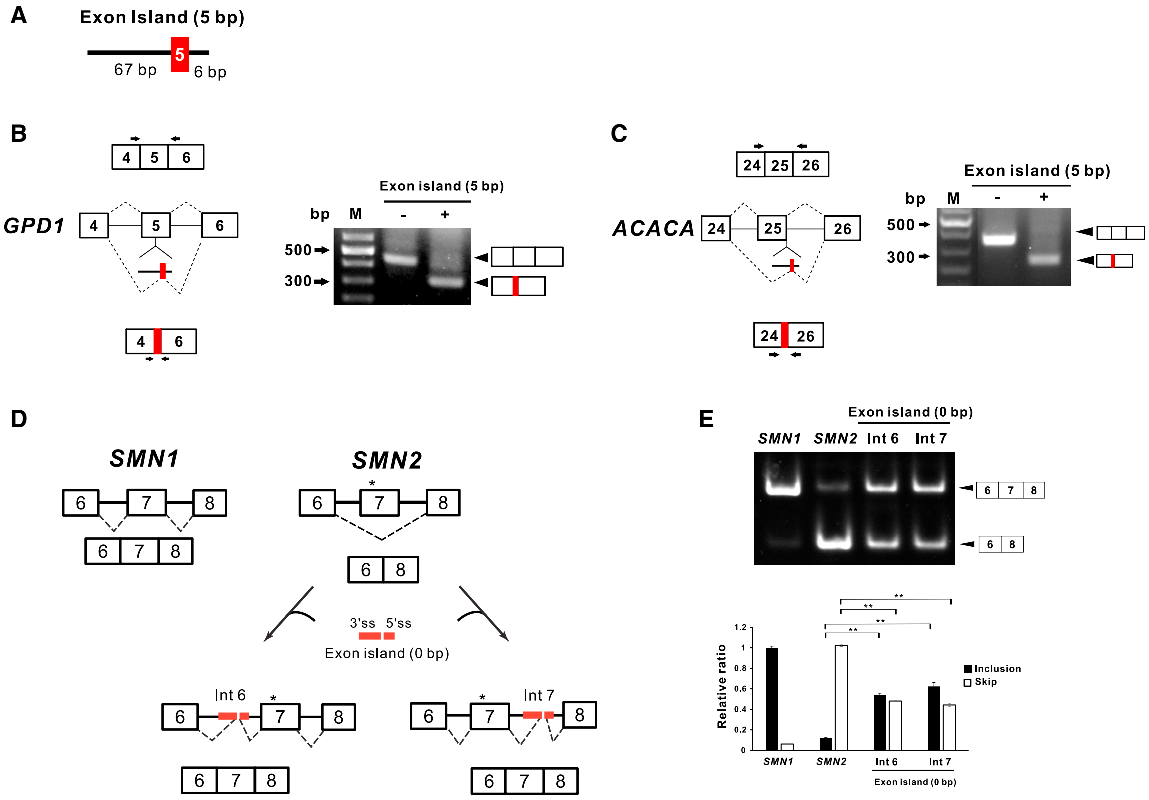

Figure 5. Exon island of *MPC1* acts as a regulator of splicing processes

(A) Structure of the exon island (5 bp) which has "TAACG" in-between 67 bp of 3′ss of *MPC1* microexon flanking upstream intron and the 6 bp 5′ss consensus. (B) Exon island (5 bp) was inserted in the middle of exon 5 (113 bp) of *GPD1* minigene construct. Primers used in RT-PCR are marked as arrows (left). Agarose gel analysis of RT-PCR products (right). (C) Exon island (5 bp) was inserted in the 5′ss of intron 25 of *ACACA* minigene construct (left). Agarose gel analysis of RT-PCR products amplified with primers marked as arrows (right). (D) and (E) Splicing modulation of *SMN2* exon7 by exon island. *SMN1* and *SMN2* minigene were placed between the SV40 promoter and *EGFP* gene in the pSV40-EGFP backbone. Asterisk (*) indicates C>T mutation in exon 7 of *SMN2*. *SMN2* minigene vector was modified by the insertion of exon island (0 bp) into either intron 6 or intron 7. The exon island (0 bp) has no exon sequence. Constructs were transfected into HeLa cells followed by total RNA extraction and RT-PCR. The RT-PCR products amplified with the primers shown in (E) were analyzed in 6% acrylamide gel electrophoresis. For evaluating the efficiency of exon 7 inclusion in final mRNA, quantitative real-time RT-PCR was performed. Primers are listed in Table S2. All bar graphs are plotted as mean ± SE. **$p < 0.01$ by Student's two-tailed t test.

designed wt *SMN1* and *SMN2* minigene constructs encompassing exons 6 to 8 of respective genes (Figure 5D). Furthermore, it was verified that C to T mutation at exon 7 of *SMN2* led to exon 7 skipping while exon 7 was included in SMN1 mRNA (Figure 5E lanes 1 and 2). To test the ability of the exon island to facilitate the inclusion of exon 7 of *SMN2*, two modified *SMN2* minigene constructs were prepared by inserting the exon island (0 bp) into either upstream (intron 6) or downstream intron (intron 7), respectively (Figure 5D). These constructs were transfected into HeLa cells, and the splicing variants from each SMN minigene were visualized by electrophoresis and quantified by real-time RT-PCR. Remarkably, the insertion of the exon island (0 bp) at intron 6 and intron 7 strongly stimulated the substantial inclusion of exon 7 in the final mRNA (Figure 5E). Further sequencing analysis revealed that no additional sequence was detected at exon junctions since the exon island (0 bp) used in this experiment did not have an exon sequence between its 3′ss and 5′ss, indicating that the exon definition of the exon island (0 bp) acts as an enhancer for potentiating neighboring exon inclusion without perturbing original sequences. Collectively, these data suggest that the exon island of *MPC1* has strong splicing-inducing strength, which can modify canonical splicing processes depending on where it is inserted.

DISCUSSION

There is growing evidence on the biological importance of microexons alongside their splicing mechanisms. However, few studies have elucidated non-3x microexon splicing. Here, we showed how non-3x microexon of *MPC1* (4 bp) was spliced through the experiments using various minigene and exon island reporters. We also found that the proximal intron region of 67 bp 3′ss and 6 bp 5′ss of the *MPC1* microexon has strong exon definitive activity and raised the possibility of its usage as a modifier for splicing processes in target genes.

Considering the reading frame, alternative splicing of non-3x bp microexons should be precisely regulated. Our observation in Figure 1B shows that non-3x bp microexons under 10 bp from five genes

exhibited constitutively spliced in the final mRNA. We obtained consistent data with differentiated and undifferentiated 3T3L1 (mouse preadipocyte) and C_2C_{12} (mouse skeletal muscle) (Figure S1). However, the transient expression in HeLa cells with artificial reporter studies did not implement constitutive splicing of microexons of *TNNI1, TMEM237, KSR1,* and *MUSK* except for the *MPC1* microexon (Figure 2F). These discrepancies in the microexon splicing pattern observed in two different experimental settings may be because the artificially shortened intron does not have sufficient intronic elements for complete microexon splicing except for the *MPC1* microexon. In addition, the lack of tissue-specific RBPs in HeLa cells might lead to incomplete inclusion of microexons of four genes except for *MPC1*, which could be supported by the fact that, unlike *MPC1*, the four genes are tissue-specifically expressed.

Despite experimental limitations with reporter constructs, the inclusions of *MPC1* microexon in mRNA seems quite consistent in various experimental settings. Usually, constitutively spliced microexons are predicted to have stronger splice-site tendencies, shorter flanking introns, and a higher ESE density than those of longer exons.[9] However, *MPC1* microexon, 4 bp, is extremely short to accommodate sufficient ESE and has long upstream and downstream flanking introns, over 12 and 3 kbp, respectively. Moreover, *MPC1* microexon is highly expected to skip because it has a high skipping score (S = 4.22) when assessed using computational methods using MaxEntScan.[25,26]

In this study, we first demonstrated that both splicing sites bordering the *MPC1* microexon have strong splicing activity sufficient to splice 4, 2, and 1 bp, and even 0 bp (Figure 3). Particularly, our studies of splicing intermediates and mutagenesis of splice sites strongly indicate that the *MPC1* microexon is spliced by its exon definition in a manner different from common exons where the exon junction complex deposited after the splicing reaction strongly inhibits recursive splicing in its vicinity,[27–30] thereby preventing cryptic splicing from producing microexons. Interestingly, our lariat studies identified several branchpoints (Figure 3E). It turned out that two “A’s” at −68 and −63 nucleotides from microexon are the major branching positions near the pyrimidine-rich region, which are unusually distant locations compared with regular branchpoints positioned at a median of 28 bp upstream of the 3′ss.[31] This distal pyrimidine-rich region might help to overcome the proximity between 3′ss and 5′ss bordering the 4 bp microexon, allowing the accurate splicing of microexons.

We also provide evidence that the 67 bp 3′ss of *MPC1* microexon flanking intron is critical for its exon definition through various reconstituted microexon and mutagenesis studies. This minimal region (67 bp 3′ss) is sufficient not only for the *MPC1* microexon but also for the correct inclusion of reconstituted microexons of *ACACA* with the exceptions of those of *GPD1* that were partially recovered (Figure 4I). Assuming that the role of 5′ss 6 bp of *MPC1* microexon on splicing is sufficient, only one nucleotide in 5′ss of *GPD1* was substituted with that of *MPC1* (guGaga > guAaga). However, the splicing-in of reconstituted *GPD1* microexons was incomplete (Figures S2B and S2C). The complete inclusion of the reconstituted *GPD1* microexon was achieved by the replacement of the 5′ss region of the *GPD1* microexon with that of the *MPC1* (27 bp), which possibly indicates that the subtle difference in the strength of 5′ss could determine the complete inclusion of microexon although exact underlying mechanisms are currently unclear.

Previous studies demonstrated that flanking introns have information that codes for the correct splicing of microexons in a variety of contexts within the cell.[5,9,13,17,18,32–34] In addition, the functional association between specific histone modifications and microexon splicing events has been elucidated.[35,36] Moreover, there are complexities in combined actions among the location and numbers of intronic *cis* elements as well as temporal- and tissue-specific availability of RBPs for the accurate splicing of microexons. There is a high sequence similarity at the upstream proximal intron between species as shown in Figure S1. The pyrimidine-rich region of *MPC1* microexons is located farther upstream than its common exons, probably to overcome spatial limitations in microexon splicing through exon definition. Direct repeat of “TGTCTG” and highly conserved sequences downstream of pyrimidine tract needs further study to elucidate the interaction of the RBPs or other regulators for splicing.

Since we verified a strong exon definition of the minimal region of *MPC1* microexon flanking introns, we presented the possibilities of using it as a modifier of genuine splicing processes (Figure 5). The target exon can be skipped during splicing by the insertion of the exon island into the target exon or splice site, maintaining the reading frame. Strikingly, we also showed that the exon island (0 bp), which has no exon sequence, positioned in intron 6 or intron 7 of *SMN2* minigene markedly enhanced exon 7 inclusion without any sequence addition or reading frameshift. The observation that the 5′ss destruction of the exon island did not enhance exon 7 inclusion strongly suggests that complete exon definition of the exon island is essential for its splicing enhancing action (Figure S4). Future investigations *in vivo* models are necessary to clarify the efficacy of the exon island (0 bp) usage regarding SMN protein expression and to compare its potency with that of other splicing-editing tools such as antisense oligonucleotide, small molecules, and targeting splicing regulating elements.[37]

Overall, we identify the possible usages of the exon island of *MPC1* in various genetic contexts. This study may provide insights for effective and precise splicing-mediated gene correction.

MATERIALS AND METHODS

Cell culture and transfection

HeLa cells were grown in Dulbecco’s modified Eagle medium containing 10% heat-inactivated fetal bovine serum, 100 unit/mL penicillin, and 100 g/mL streptomycin. For ViaFect (Promega, Madison, WI) was used in transient expression according to the manufacturer’s introductions.

Generation of minigene constructs

MPC1 minigene construct containing its microexon (exon 2) is constructed by the insertion of the 3′ region of exon 1 to 5′ region of

intron 1 (901 bp) (chr6: 166,381,901-166,382,878), 3′ region of intron 1 (329 bp) to 5′ region of intron 2 (541bp) (chr6: 166,369,677-166,370,544), and 3′ region of intron 2 (259 bp) to 5′ region of exon 3 (496b0) (chr6: 166,366,843-166,366,794) in order between the SV40 promoter and the EGFP gene of pSV40-EGFP backbone. MPC1 Swap-1/3 and 2/3 constructs are prepared by replacing the intron 1 and intron 2 with intron 3 (688 bp) (chr6: 166,366,107-166,370,544), respectively. QuickChange reaction was performed using the amplicon of intron 3 of which both ends contain the sequences of either side of the site to be inserted. MPC1 hybrid-3/1 and 1/3 constructs are prepared by replacing the 5′ region of intron 1 (901 bp) with the 5′ region of intron 3 (349 bp) (chr6: 166,366,446-166,366,794) and the 3′ region of intron 1 (329 bp) with the 3′ region of intron 3 (339 bp) (chr6: 166,366,107-166,366,445) in *MPC1* minigene construct, respectively. Hybrid-3/1 serial deletion constructs and *MPC1* minigene constructs containing different lengths (0, 1, 2, and 3 bp) of microexon were generated by QuickChange reaction using the respective pair of primers (Table S1).

Generation of "exon island" reporter constructs

Plasmid SV40-EGFP backbone is constructed by the ligation of SV40 promoter, *EGFP* gene without ATG start codon, followed by SV40 poly(A) signal sequence. For microexon island construction, the amplicon of *MPC1* gene from the 3′ region of exon 3 to the 5′ region of exon 4 (hg38:chr6: 166,366,102-166,366,809) was inserted between the SV40 promoter and the *EGFP* gene. Then, ATG start codon having Kozak sequence and three kinds of the reading frame was made in *MPC1* exon 3 region and EcoRV restriction site was introduced at the middle of intron 3 (chr6: 166366432) (pSV40-EGFPa, b, c). Microexon island constructs were prepared by insertion of the microexon and both flanking regions of *TNNI1* (chr1: 201,416,899-201,417,360), *KSR1* (chr17: 27,585,451-853), *TMEM237* (chr2: 201, 639,991-201,640,463), and *MUSK* (chr9: 110,762,013-110,762,442) at EcoRV site.

Generation of reconstituted microexon minigene constructs

GPD1 minigene construct containing exon 6 is constructed by the insertion of the 3′ region of exon 5 to the 5′ region of exon 7 (hg38; chr17: 50,106,394-50,107,573) between the SV40 promoter and the EGFP gene of pSV40-EGFP backbone. *GPD1* microexon vectors are generated by QuickChange Kit using the forward and reverse primers of GPD-20, GPD8, and GPD-5 (Table S1). To replace intron 5 3′ss in *GPD1* minigene constructs with 96 bp of *MPC1* intron 1 3′ss, *MPC1* intron 1 3′ss region was amplified using GPD-3ss-fwd and respective reverse primers (GPD-113-3ss-rev, GPD-20-3ss-rev, GPD-8-3ss-rev, and GPD-5-3ss-rev), and PCR products were used in QuickChange reactions. Modification of 3′ss and 5′ss in *GPD1* minigene constructs was done by QuickChange with the primers listed in Table S1 and respective GPD 3′ss constructs as used as a template for PCR. *ACACA* minigene construct containing exon 25 is generated by the insertion of the 3′ region of exon 24 to the 5′ region of intron 24 (hg38; chr17: 37,240,524-37,240,230), 3′ region of intron 24 to 5′ region of intron 25 (chr17: 37,235,326-37,234,785), and 3′ region of intron 25 to 5′ region of exon 26 (Chr17: 37,226,641-37,226,421) in order between the SV40 promoter and the EGFP gene of pSV40-EGFP backbone.

Generation of *SMN1* and *SMN2* minigene constructs

SMN1 and *SMN2* minigene construct containing exon 7 is produced by the insertion of the 3′ region of exon 6 to the 5′ region of intron 6 (hg38; chr5: 70,946,149-70,946,593 and hg38; chr5: 70,070,724-70,071,168, respectively), and 3′ region of intron 6 to 5′ region of exon 8 (chr5: 70,951,501-70,952,510 and chr5: 70,076,081-70,077,090, respectively) in order between the SV40 promoter and the EGFP gene in pSV40-EGFP backbone.

Measurement of splicing activity

We transiently transfected the minigene construct into HeLa cells. After 48 h of transfection, whole-cell lysates were prepared with 1x Passive lysis buffer (Promega, Madison, WI, USA). EGFP and mCherry intensities were measured with supernatant after centrifugation at 8,000 $\times g$ (TECAN, infinite F200 pro, Männedorf, Switzerland). The value of GFP intensity was divided by mCherry intensity for normalization according to transfection efficiency.

RNA extraction and quantitative real-time RT-PCR

For quantitative real-time RT-PCR (qPCR), cDNAs were synthesized from 4 μg of total RNA using random hexamer primers and SuperScript reverse transcriptase III (Themo Fisher Scientific, Waltham, MA) following the manufacturer's instructions. Diluted cDNAs were used as a template for qPCR using the primers (Table S2) with the SYBR Green Master Mix (Applied Biosystems; Foster City, CA). Reactions are performed using the ABI PRISM 7300 RT-PCR System (Applied Biosystems).

Analysis of lariats

Ten micrograms of total RNA was treated with ribonuclease R (Biosearch Technologies, Hoddesdon, UK) for 1 h at 37°C and purified with Monarch RNA extraction kit (New England Biolabs, Ipswich, MA). First PCR was done with Super-Script IV One-step RT-PCR system (Themo Fisher Scientific, Waltham, MA). The primers were as follows: lariat "a" forward (f) 5′-CAGGTGTGAGGTTG-CAGTAACCT-3′ and reverse (r) 5′-GACACAGTC-TTCAGTTCAGTGTGC-3'; lariat "b" (f) 5′- GACTGCTGAATATCTGACAGCG-3′ and (r) 5′-AGGAG CATGGCTGTCACAGA-3′; lariat "c" (f) 5′-GACTGCTGAATATCT GACAG-CG-3′, (r) 5′-CACTCTTCCATGCCATCACATTCTGTAT GT-3′. Second PCR was done using LaboPass IP-*Taq* DNA Polymerase (Cosmogenetech, Seoul, South Korea). One-10th diluted first PCR products were used as PCR templates. The primers were as follows: lariat "a" primer (f) 5′-GTTGCAGTAACCTAATAAGACCAA-3′ and (r) 5′-CACTCTTCCATG-CCATCACATTCTGTATGT-3'; lariat "b" (f) 5′- CCAAACCCTTTCTAGCTCTG-3′, (r) 5′- TGCAGTAGGC ACCCTTCACA-3'; lariat "c" (f) 5′-AGGAGCATGGCTGTCACAGA-3′ and (r) 5′-TCTTGAAATGCAAGCAGGAGC-3′. PCR products are cloned into pBluescript vector followed by sequencing to analyze branchpoints.

Statistical analysis

All results are expressed as the mean ± standard error (SE). The Student's t test was used for comparison between two groups. A p value < 0.05 was considered statistically significant.

DATA AVAILABILITY

The data that support the findings of this study are available in the supplemental information of this article.

SUPPLEMENTAL INFORMATION

Supplemental information can be found online at https://doi.org/10.1016/j.omtn.2023.01.010.

ACKNOWLEDGMENTS

This work was supported by the National Research Foundation of Korea (NRF) grant funded by the Korean Government (MSIT) [NRF-2020R1A2C1004833, NRF-2017R1A2B4007462, NRF-2017R1A2B4009674]. This work was supported by a faculty research grant of Yonsei University College of Medicine (6-2022-0063). Funding for open access charge: National Research Foundation of Korea.

AUTHOR CONTRIBUTIONS

E.K. and K.K. conceived and designed the experiments. E.K. and D.S. conducted most of the cell and biochemical experiments. K.K. performed bioinformatics analyses. E.K. and K.K. analyzed and interpreted results and wrote the manuscript with input from all authors. All authors contributed to editing the manuscript.

DECLARATION OF INTERESTS

The authors declare no competing interests.

REFERENCES

1. Ule, J., and Blencowe, B.J. (2019). Alternative splicing regulatory networks: functions, mechanisms, and evolution. Mol. Cell *76*, 329–345.
2. Scotti, M.M., and Swanson, M.S. (2016). RNA mis-splicing in disease. Nat. Rev. Genet. *17*, 19–32.
3. Hawkins, J.D. (1988). A survey on intron and exon lengths. Nucleic Acids Res. *16*, 9893–9908.
4. Berget, S.M. (1995). Exon recognition in vertebrate splicing. J. Biol. Chem. *270*, 2411–2414.
5. Dominski, Z., and Kole, R. (1991). Selection of splice sites in pre-mRNAs with short internal exons. Mol. Cell Biol. *11*, 6075–6083.
6. Black, D.L. (1991). Does steric interference between splice sites block the splicing of a short c-src neuron-specific exon in non-neuronal cells? Genes Dev. *5*, 389–402.
7. Volfovsky, N., Haas, B.J., and Salzberg, S.L. (2003). Computational discovery of internal micro-exons. Genome Res. *13*, 1216–1221.
8. Wu, T.D., and Watanabe, C.K. (2005). GMAP: a genomic mapping and alignment program for mRNA and EST sequences. Bioinformatics *21*, 1859–1875.
9. Li, Y.I., Sanchez-Pulido, L., Haerty, W., and Ponting, C.P. (2015). RBFOX and PTBP1 proteins regulate the alternative splicing of micro-exons in human brain transcripts. Genome Res. *25*, 1–13.
10. Capponi, S., Stöffler, N., Irimia, M., Van Schaik, F.M.A., Ondik, M.M., Biniossek, M.L., Lehmann, L., Mitschke, J., Vermunt, M.W., Creyghton, M.P., et al. (2020). Neuronal-specific microexon splicing of TAF1 mRNA is directly regulated by SRRM4/nSR100. RNA Biol. *17*, 62–74.
11. Lin, L., Zhang, M., Stoilov, P., Chen, L., and Zheng, S. (2020). Developmental attenuation of neuronal apoptosis by neural-specific splicing of Bak1 microexon. Neuron *107*, 1180–1196.e8.
12. Head, S.A., Hernandez-Alias, X., Yang, J.S., Ciampi, L., Beltran-Sastre, V., Torres-Méndez, A., Irimia, M., Schaefer, M.H., and Serrano, L. (2021). Silencing of SRRM4 suppresses microexon inclusion and promotes tumor growth across cancers. PLoS Biol. *19*, e3001138.
13. Gonatopoulos-Pournatzis, T., Wu, M., Braunschweig, U., Roth, J., Han, H., Best, A.J., Raj, B., Aregger, M., O'Hanlon, D., Ellis, J.D., et al. (2018). Genome-wide CRISPR-cas9 interrogation of splicing networks reveals a mechanism for recognition of autism-misregulated neuronal microexons. Mol. Cell *72*, 510–524.e12.
14. Lee, J., Villarreal, O.D., Chen, X., Zandee, S., Young, Y.K., Torok, C., Lamarche-Vane, N., Prat, A., Rivest, S., Gosselin, D., et al. (2020). QUAKING regulates microexon alternative splicing of the Rho GTPase pathway and controls microglia homeostasis. Cell Rep. *33*, 108560.
15. Irimia, M., Weatheritt, R.J., Ellis, J.D., Parikshak, N.N., Gonatopoulos-Pournatzis, T., Babor, M., Quesnel-Vallières, M., Tapial, J., Raj, B., O'Hanlon, D., et al. (2014). A highly conserved program of neuronal microexons is misregulated in autistic brains. Cell *159*, 1511–1523.
16. Li, Y., Zhang, Q., Lovnicki, J., Chen, R., Fazli, L., Wang, Y., Gleave, M., Huang, J., and Dong, X. (2019). SRRM4 gene expression correlates with neuroendocrine prostate cancer. Prostate *79*, 96–104.
17. Carlo, T., Sierra, R., and Berget, S.M. (2000). A 5' splice site-proximal enhancer binds SF1 and activates exon bridging of a microexon. Mol. Cell Biol. *20*, 3988–3995.
18. Dominski, Z., and Kole, R. (1992). Cooperation of pre-mRNA sequence elements in splice site selection. Mol. Cell Biol. *12*, 2108–2114.
19. Lykke-Andersen, S., and Jensen, T.H. (2015). Nonsense-mediated mRNA decay: an intricate machinery that shapes transcriptomes. Nat. Rev. Mol. Cell Biol. *16*, 665–677.
20. Bricker, D.K., Taylor, E.B., Schell, J.C., Orsak, T., Boutron, A., Chen, Y.C., Cox, J.E., Cardon, C.M., Van Vranken, J.G., Dephoure, N., et al. (2012). A mitochondrial pyruvate carrier required for pyruvate uptake in yeast, Drosophila, and humans. Science *337*, 96–100.
21. Herzig, S., Raemy, E., Montessuit, S., Veuthey, J.L., Zamboni, N., Westermann, B., Kunji, E.R.S., and Martinou, J.C. (2012). Identification and functional expression of the mitochondrial pyruvate carrier. Science *337*, 93–96.
22. Koh, E., Kim, Y.K., Shin, D., and Kim, K.S. (2018). MPC1 is essential for PGC-1α-induced mitochondrial respiration and biogenesis. Biochem. J. *475*, 1687–1699.
23. Piovesan, A., Antonaros, F., Vitale, L., Strippoli, P., Pelleri, M.C., and Caracausi, M. (2019). Human protein-coding genes and gene feature statistics in 2019. BMC Res. Notes *12*, 315.
24. Lefebvre, S., Bürglen, L., Reboullet, S., Clermont, O., Burlet, P., Viollet, L., Benichou, B., Cruaud, C., Millasseau, P., Zeviani, M., et al. (1995). Identification and characterization of a spinal muscular atrophy-determining gene. Cell *80*, 155–165.
25. Ye, Z., Chen, Z., Lan, X., Hara, S., Sunkel, B., Huang, T.H.M., Elnitski, L., Wang, Q., and Jin, V.X. (2014). Computational analysis reveals a correlation of exon-skipping events with splicing, transcription and epigenetic factors. Nucleic Acids Res. *42*, 2856–2869.
26. Yeo, G., and Burge, C.B. (2004). Maximum entropy modeling of short sequence motifs with applications to RNA splicing signals. J. Comput. Biol. *11*, 377–394.
27. Pai, A.A., Paggi, J.M., Yan, P., Adelman, K., and Burge, C.B. (2018). Numerous recursive sites contribute to accuracy of splicing in long introns in flies. PLoS Genet. *14*, e1007588.
28. Joseph, B., Kondo, S., and Lai, E.C. (2018). Short cryptic exons mediate recursive splicing in Drosophila. Nat. Struct. Mol. Biol. *25*, 365–371.
29. Blazquez, L., Emmett, W., Faraway, R., Pineda, J.M.B., Bajew, S., Gohr, A., Haberman, N., Sibley, C.R., Bradley, R.K., Irimia, M., et al. (2018). Exon junction complex shapes the transcriptome by repressing recursive splicing. Mol. Cell *72*, 496–509.e9.
30. Boehm, V., Britto-Borges, T., Steckelberg, A.L., Singh, K.K., Gerbracht, J.V., Gueney, E., Blazquez, L., Altmüller, J., Dieterich, C., and Gehring, N.H. (2018). Exon junction complexes suppress spurious splice sites to safeguard transcriptome integrity. Mol. Cell *72*, 482–495.e7.

31. Pineda, J.M.B., and Bradley, R.K. (2018). Most human introns are recognized via multiple and tissue-specific branchpoints. Genes Dev. *32*, 577–591.

32. Black, D.L. (1992). Activation of c-src neuron-specific splicing by an unusual RNA element in vivo and in vitro. Cell *69*, 795–807.

33. Carlo, T., Sterner, D.A., and Berget, S.M. (1996). An intron splicing enhancer containing a G-rich repeat facilitates inclusion of a vertebrate micro-exon. RNA *2*, 342–353.

34. Modafferi, E.F., and Black, D.L. (1997). A complex intronic splicing enhancer from the c-src pre-mRNA activates inclusion of a heterologous exon. Mol. Cell Biol. *17*, 6537–6545.

35. Luco, R.F., Pan, Q., Tominaga, K., Blencowe, B.J., Pereira-Smith, O.M., and Misteli, T. (2010). Regulation of alternative splicing by histone modifications. Science *327*, 996–1000.

36. Hu, Q., Kim, E.J., Feng, J., Grant, G.R., and Heller, E.A. (2017). Histone posttranslational modifications predict specific alternative exon subtypes in mammalian brain. PLoS Comput. Biol. *13*, e1005602.

37. Nurputra, D.K., Lai, P.S., Harahap, N.I.F., Morikawa, S., Yamamoto, T., Nishimura, N., Kubo, Y., Takeuchi, A., Saito, T., Takeshima, Y., et al. (2013). Spinal muscular atrophy: from gene discovery to clinical trials. Ann. Hum. Genet. *77*, 435–463.

OMTN, Volume *31*

Supplemental information

Exon definitive regions for *MPC1* microexon splicing and its usage for splicing modulation

Eunjin Koh, Daye Shin, and Kyung-Sup Kim

Table_S1

Primers used in reporter construct generation

primer	Sequence (5' to 3')
Int31-199-s	GAAACTGTCATTAAACTGACTTGAGTTCTTATTAGAAATAATATTACTTGATC
Int31-199-r	GTAATATTATTTCTAATAAGAACTCAAGTCAGTTTAATGACAGTTTCCCCCAAA
Int31-96-s	GGAAACTGTCATTAAACTGACTTGGTTGCAGTAACCTAATAAGACCAAAATCAT
Int31-96-r	TGGTCTTATTAGGTTACTGCAACCAAGTCAGTTTAATGACAGTTTCCCCCAAA
Int31-67-s	GGAAACTGTCATTAAACTGACTTGTTCCATGTCTGTCCTGTCTGAACTTCGGTG
Int31-67-r	GTTCAGACAGGACAGACATGGAACAAGTCAGTTTAATGACAGTTTCCCCCAAA
Int31-46-s	CTGTCATTAAACTGACTTGTTTGATTTTTGTTTCTTTTTTTTCCATGTCTGTCCTGTCT
Int31-46-r	GACATGGAAAAAAAAGAAACAAAAATCAAACAAGTCAGTTTAATGACAGTTTCCCCCAAA
Int31-25-s	GAAACTGTCATTAAACTGACTTGCTTCGGTGACTCTTCACAAAATAGTACGGTA
Int31-25-r	CTATTTTGTGAAGAGTCACCGAAGCAAGTCAGTTTAATGACAGTTTCCCCCAAA
ex-4d4-FOR	GTGACTCTTCACAAAATAGGTAAGAAAATTATTTCCATCCATTT
ex-4d4-REV	ATGGAAATAATTTTCTTACCTATTTTGTGAAGAGTCACCGAAGT
ex-3d4-FOR	GTGACTCTTCACAAAATAGGGTAAGAAAATTATTTCCATCCATTT
ex-3d4-REV	AAATGGATGGAAATAATTTTCTTACCCTATTTTGTGAAGAGTCAC
ex-2d4-FOR	GTGACTCTTCACAAAATAGTGGTAAGAAAATTATTTCCATCCATTT
ex-2d4-REV	ATGGAAATAATTTTCTTACCACTATTTTGTGAAGAGTCACCGAAGT
ex-1d4-FOR	GTGACTCTTCACAAAATAGTCGGTAAGAAAATTATTTCCATCCATTT
ex-1d4-REV	ATGGAAATAATTTTCTTACCGACTATTTTGTGAAGAGTCACCGAAGT
b-globin-intron-fwd	TTATTTGTCAGATTGTAAGTGTTGCAGTAACCTAATAAGACC
b-globin-intron-rev	AAAAGTGATTAGAGAAAGTTCTTACCGTACTATTTTGTGAAGA
EF1a-intron-fwd	GCACCTCGATTAGTTCTCGAGTTGCAGTAACCTAATAAGACC
EF1a-intron-rev	GACGTACTCCAAAAGCTCGATCTTACCGTACTATTTTGTGAAGA
GPD-20-fwd	CTTTAGGCTGCAAGGACCCGGCCCAGGTGAGAGGGGCACAGAGGCAGCTATGG
GPD-20-rev	CCATAGCTGCCTCTGTGCCCCTCTCACCTGGGCCGGGTCCTTGCAGCCTAAAG
GPD-8-fwd	CCCTCCTCACTTTAGGCTGCAAGGTGAGAGGGGCACAGAGGCAGCTATG
GPD-8-rev	CATAGCTGCCTCTGTGCCCCTCTCACCTTGCAGCCTAAAGTGAGGAGGG
GPD-5-fwd	AGCCTTGCCCCCTCCTCACTTTAGGCAAGGTGAGAGGGGCACAGAGGCAGCTATG
GPD-5-rev	CATAGCTGCCTCTGTGCCCCTCTCACCTTGCCTAAAGTGAGGAGGGGGCAAGGCT
GPD-3ss-fwd	TAAGCCCAGGAGTTTGAGTCCAGTTGCAGTAACCTAATAAGACC
GPD-113-3ss-rev	TCCCTGGGCCGGGTCCTTGCAGCCTATTTTGTGAAGAGTCACCGAAG
GPD-20-3ss-rev	CTCACCTGGGCCGGGTCCTTGCAGCCTATTTTGTGAAGAGTCACCGAAGTT
GPD-8-3ss-rev	CTCTGTGCCCCTCTCACCTTGCAGCCTATTTTGTGAAGAGTCACCGAAGT

GPD-5-3ss-rev	TGCCTCTGTGCCCCTCTCACCTTGCCTATTTTGTGAAGAGTCACCGAAG
GPD-113-35ss-fwd	GGAGCCTTAAAGGTAAGAAAATTATTTCCATCCATTTCAGGTGAGGAGAAGGCCCCAAAG
GPD-113-35ss-rev	TTCTCCTCACCTGAAATGGATGGAAATAATTTTCTTACCTTTAAGGCTCCACAGATCTCT
GPD-20-35ss-fwd	CCCGGCCCAGGTAAGAAAATTATTTCCATCCATTTCAGGTGAGGAGAAGGCCCCAAAG
GPD-20-35ss-rev	TTCTCCTCACCTGAAATGGATGGAAATAATTTTCTTACCTGGGCCGGGTCCTTGCAGC
GPD-8-35ss-fwd	AAATAGGCTGCAAGGTAAGAAAATTATTTCCATCCATTTCAGGTGAGGAGAAGGCCCCAAAG
GPD-8-35ss-rev	TTCTCCTCACCTGAAATGGATGGAAATAATTTTCTTACCTTGCAGCCTATTTTGTGAAGAGT
GPD-5-35ss-fwd	ACAAAATAGGCAAGGTAAGAAAATTATTTCCATCCATTTCAGGTGAGGAGAAGGCCCCAAAG
GPD-5-35ss-rev	TTCTCCTCACCTGAAATGGATGGAAATAATTTTCTTACCTTGCCTATTTTGTGAAGAGTCAC
GPD-8-3ss-5ssmut-fwd	CTTCACAAAATAGGCTGCAAGGTAAGAGGGGCACAGAGGCAGCTATG
GPD-8-3ss-5ssmut-rev	CATAGCTGCCTCTGTGCCCCTCTTACCTTGCAGCCTATTTTGTGAAG

Table_S2

Primers used in quantitative real-time RT-PCR and conventional RT-PCR

primer	Sequence (5’ to 3’)
E1E2-s	ATTTCCGGGACTACCTCATGAGTACG
AS	GCAATGGGAAGACCCCAGTT
E1E3-s1	GATTTCCGGGACTACCTCATGAGCACT
GPD1-I-s	TTCTGTGAGACAACCATTGGCAAG
GPD1-I-as	GAACTTGTGGCCGTTTACGT
GPD1-S-s	TTCTGTGAGACAACCATTGAATGT
GPD1-S-as	GAACTTGTGGCCGTTTACGT
UE-s	GGACTATGTCCGAAGCAAGGAT
EGFP-as	TGAACAGCTCCTCGCCCTT
EGFP1-s	GTGAGCAAGGGCGAGGAGCTGTTC
EGFP1-as	TTGCCGGTGGTGCAGATGAACTTCA
E3-as	GCAATGGGAAGACCCCAGTT
I3-s	TCCTGTCTGAACTTCGGTGA
SMN_678_S	GCTATCATACTGGCTATTATATGGGTTT
SMN_68_S	GCTATCATACTGGCTATTATATGGAAATG
SMN2ex8 as	CGCTTCACATTCCAGATCTG

Figure S1

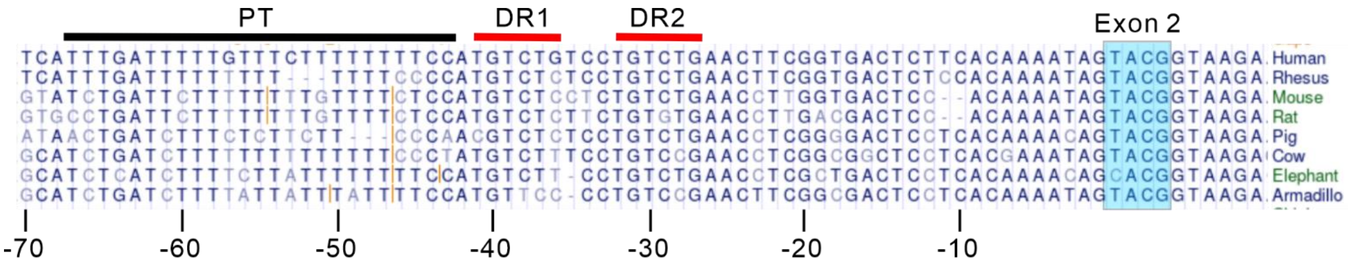

Figure S1. Sequence homology between mammalian species around the *MPC1* microexon

Figure S2

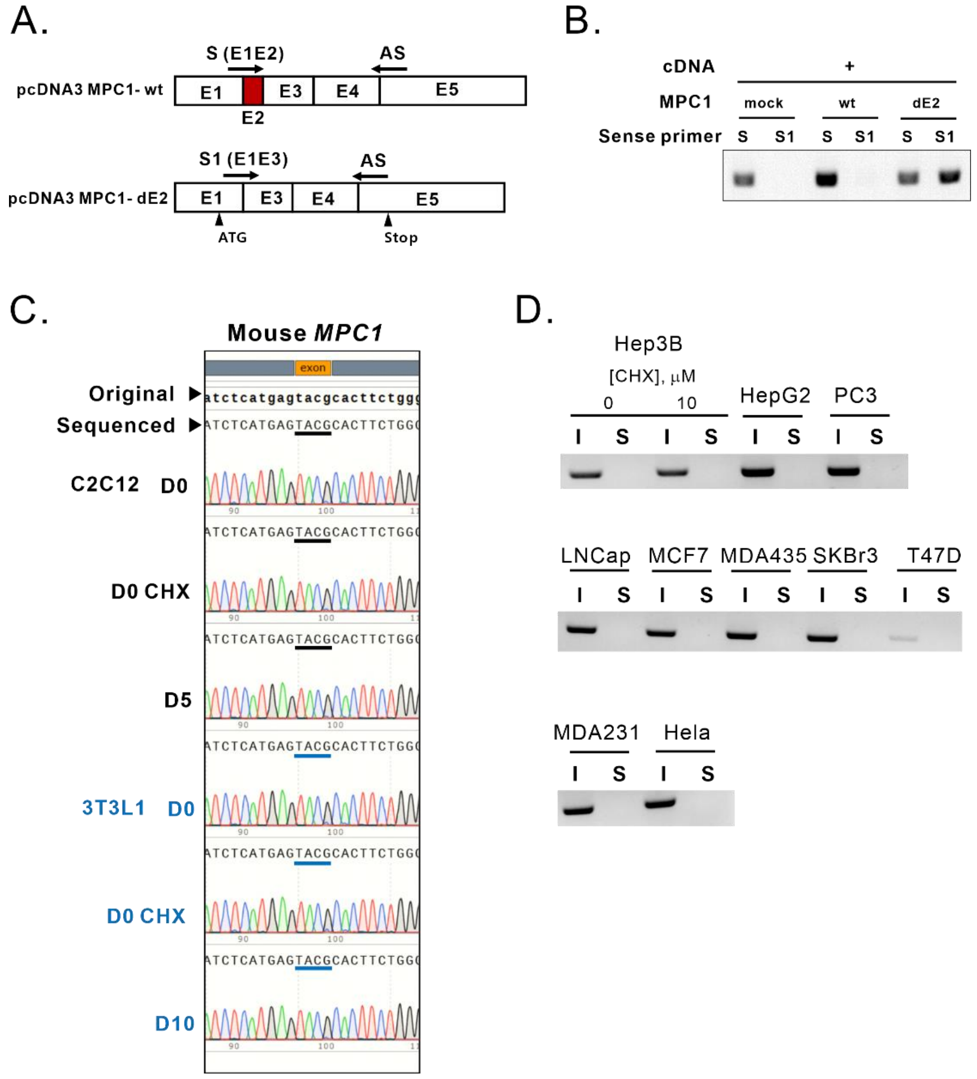

Figure S2. Detection of *MPC1* microexon-included and -skipped isoforms. (A) The primers were designed to detect the microexon skipped isoforms. The specificity was verified with the *MPC1* cDNA construct of wild-type (wt) and that of exon2 deleted (dE2) forms. (B) Agarose gel electrophoresis of RT-PCR products. Mixture of the one μl of one-fifth diluted cDNA synthesized from total RNA of 786-O cells and 10 pg of each mock, pcDNA3-MPC1-wt and pcDNA3-MPC1-dE2 were utilized as the template for PCR reactions. (C) Sequencing analysis of RT-PCR products of the mouse MPC1 mRNA prepared from C2C12 and 3T3L1 cell lines (D0, undifferentiated; D5 or D10 indicate 5 days or 10 days differentiated, respectively). Ten μM of cycloheximide (CHX) was treated for 6 hrs. Primers used in RT-PCR are mMPC-s 5'- ccacagcggtgtcatctgtc -3' and mMPC1-as 5'- gtaccgctatgcagatggcc-3'. (D) Detection of human MPC1 microexon-included or -skipped mRNA by RT-PCR in various cell lines. 'I' and 'S' indicate microexon- included and -skipped, respectively.

Figure S3

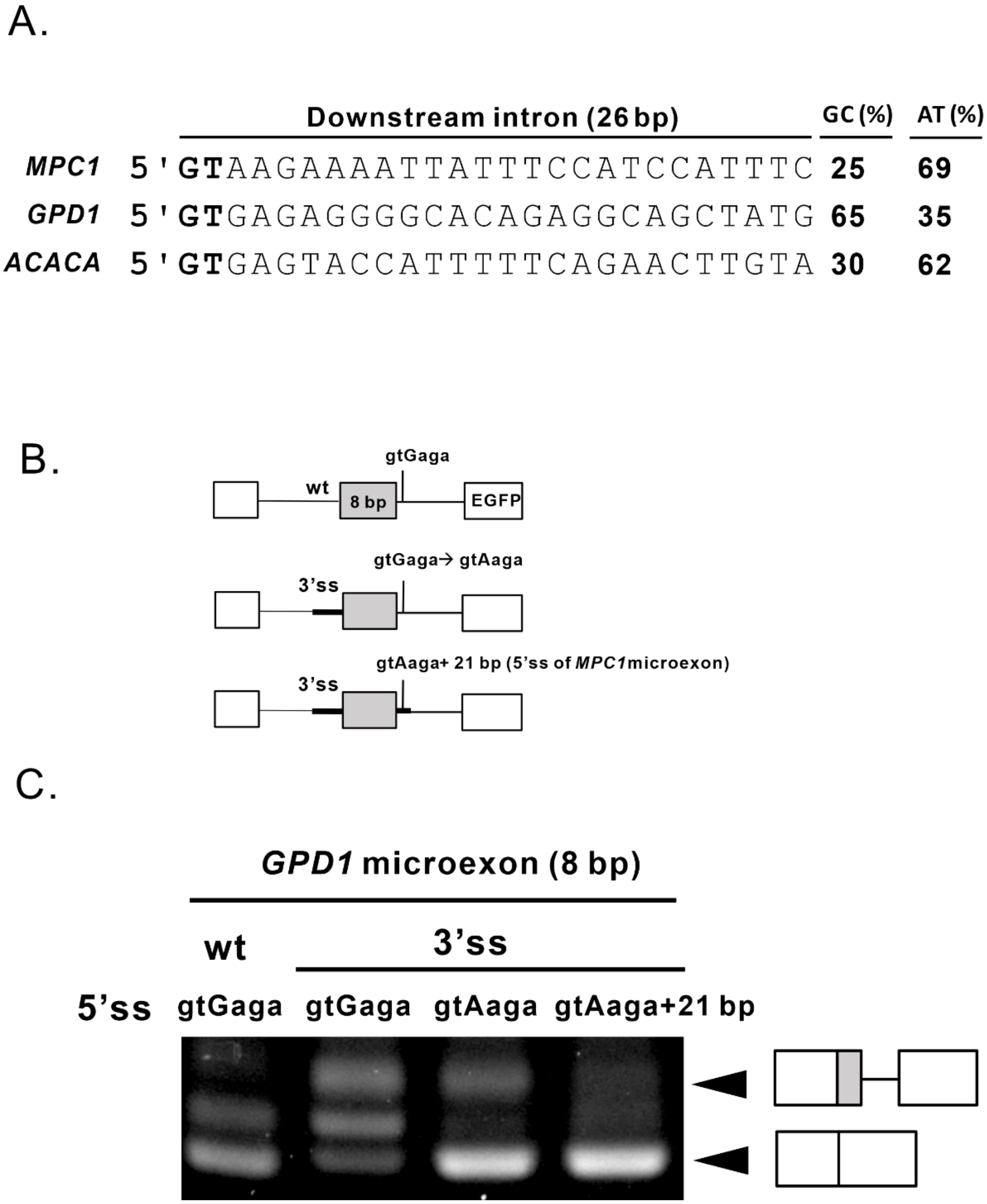

Figure S3. Cryptic GT usage is suppressed by 3'ss (96 bp) and 5'ss (25 bp) of *MPC1* microexon flanking introns. (A) Sequence alignment of 26 bp downstream intron of each microexon. Intron 2, intron 6, and intron 26 of *MPC1, GPD1*, and *ACACA*, respectively. Y; pyrimidines. (B) 5'ss of *GPD1* was mutated to that of *MPC1* (guGaga>guAaga) and was switched to 5'ss of *MPC1* microexon downstream intron (25 bp). (C) Agarose gel electrophoresis of RT-PCR products.

Figure S4

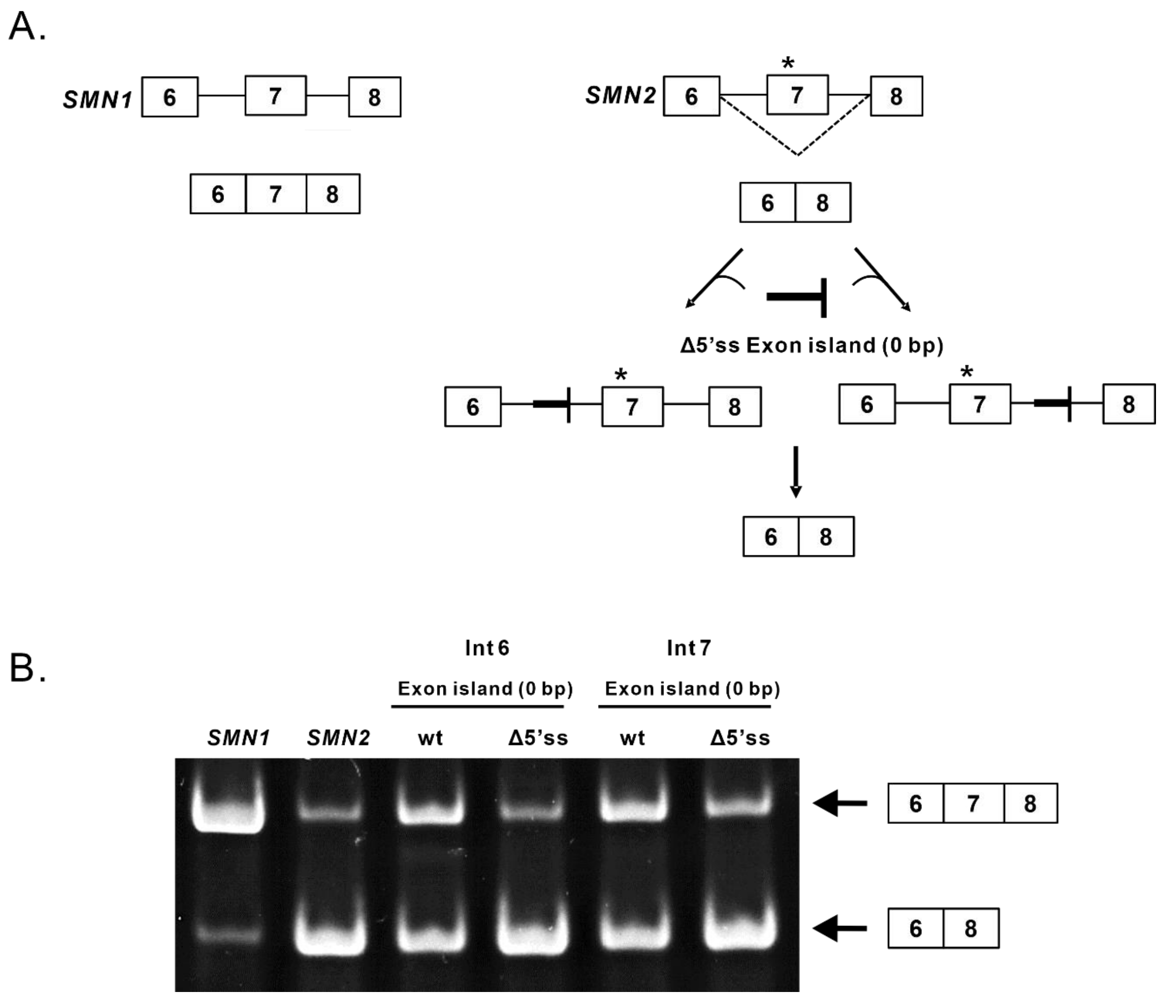

Figure S4. Insertion of the Δ5'ss exon island (0 bp) did not enhance exon 7 inclusion. (A) *SMN1* and *SMN2* minigene constructs were positioned between the SV40 promoter and *EGFP* gene in the pSV40-EGFP backbone. Asterisk (*) indicates C>T mutation. *SMN2* minigene vector was modified by the insertion of Δ5'ss exon island (0 bp) into either intron 6 or intron 7. (B) Acrylamide gel electrophoresis of RT-PCR products amplified with sense and tisense primers bind to exon 6 and exon 8, respectively.
